# Antihyperuricemic Effect of Hawthorn Flavonoid Vitexin: LC–MS Analysis, Network Pharmacology, and In Vivo Verification of Molecular Mechanisms

**DOI:** 10.1002/fsn3.71925

**Published:** 2026-05-27

**Authors:** Shu‐Yu Wu, Kai‐Wen Kang, Dan‐Yu Huang, Shu‐Ying Li, Hao Zheng, Ri‐Hui Wu, Hui‐Qing Lv, Wan‐Bin Song, Jing Chen, Cheng‐Ping Wen, Li‐She Gan, Xiao Yuan

**Affiliations:** ^1^ School of Pharmaceutical Sciences Zhejiang Chinese Medical University Hangzhou China; ^2^ School of Pharmacy and Food Engineering International Healthcare Innovation Institute, Wuyi University Jiangmen China; ^3^ State Key Laboratory of Quality Research in Chinese Medicine Macau University Science and Technology Macao China; ^4^ Department of Endocrinology The First Affiliated Hospital of Zhejiang Chinese Medical University (Zhejiang Provincial Hospital of Chinese Medicine) Hangzhou China

**Keywords:** ABCG2, hyperuricemia, inflammation, network pharmacology, vitexin

## Abstract

This study identified vitexin as one of the primary active flavonoids in hawthorn that ameliorate hyperuricemia by targeting the PPARγ/ABCG2 pathway. UPLC–MS/MS analysis indicated 17 flavonoid structures in total flavonoids of hawthorn (TFH), while SwissADME calculation then suggested vitexin as the prime candidate for further mechanistic and pharmacodynamic validation. Network pharmacology and molecular docking predicted PPARγ/ABCG2 as one of the key pathways for these flavonoids, which was confirmed by favorable binding with both PPARγ and ABCG2 and stable complex formation of vitexin with PPARγ in molecular dynamics studies. This hypothesis was validated in an HUA mouse model. Vitexin treatment effectively reduced serum uric acid by inhibiting hepatic XOD and ADA activities, and more notably, markedly upregulating renal and intestinal ABCG2 expression to near‐normal levels, along with modulation of OAT1 and GLUT9, thereby significantly enhancing urate excretion. The compound also alleviated HUA‐induced liver, kidney, and intestinal injuries, restored epithelial barrier integrity, and reduced oxidative stress and inflammation. These findings demonstrate that vitexin exerts multi‐target protective effects against HUA by simultaneously suppressing urate production, enhancing its excretion, and mitigating tissue damage. This mechanism of action is consistent with the predicted pathways and supports its potential as a natural therapeutic candidate.

## Graphical abstract

UPLC‐MS/MS analysis of hawthorn flavonoids combined with network pharmacology, molecular docking, and molecular dynamics studies predicted vitexin as a key compound acting on the PPARγ/ABCG2 pathway. In a hyperuricemic mouse model, vitexin effectively lowered serum uric acid by enhancing renal and intestinal urate excretion, inhibiting urate production, and mitigating associated tissue damage and inflammation.

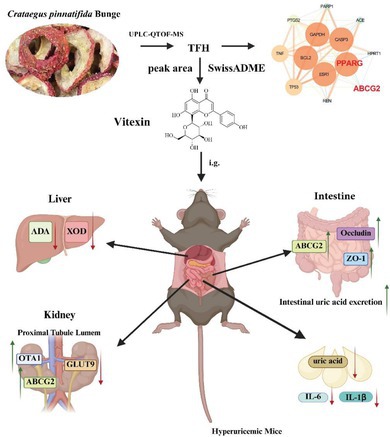

## 1 Introduction

Hyperuricemia (HUA) is a metabolic disorder characterized by either the overproduction or underexcretion of uric acid (UA). Clinically, hyperuricemia is defined as serum UA concentrations ≥ 7.0 mg/dL (416.0 μmol/L) in men and ≥ 6.0 mg/dL (357.0 μmol/L) in women (Du et al. 2024). This condition is a well‐established risk factor for various chronic diseases, including hypertension, diabetes, metabolic syndrome, renal impairment, and cardiovascular disorders (Feig et al. 2008; Muszyński et al. 2023). With improving living standards, increased consumption of purine‐rich foods, sugar‐sweetened beverages, and alcohol has contributed to a rising prevalence of HUA among adults, currently estimated at 8.4%–13.3% in China (Piao et al. 2022). This trend underscores the importance of continuous monitoring and effective management of serum UA levels to mitigate associated health risks (Gliozzi et al. 2016).

The pathogenesis of HUA primarily stems from an imbalance between UA production and excretion. On the production side, approximately one‐third of UA originates from dietary purines by adenosine deaminase (ADA) and xanthine oxidase (XOD) in the liver, while two‐thirds result from endogenous purine metabolism. Regarding excretion, under physiological conditions, about 70% of UA is eliminated by the kidneys and 30% via the intestines, and this process is regulated by various transporters, such as URAT1, GLUT9, ABCG2, and OATs (Komori et al. 2018). Pathologically, HUA can arise from hepatic overproduction of UA, or more commonly, from impaired excretion due to renal dysfunction, inherited metabolic abnormalities, or factors associated with metabolic syndrome. Furthermore, this delicate balance can be disrupted by pharmacological agents or functional declines in the renal or gastrointestinal systems, ultimately leading to disrupted UA homeostasis (Yin et al. 2022). If left inadequately controlled, chronic HUA may progress to complications such as gout, urate nephropathy, and joint deformities.

Although clinical drugs targeting the above enzymes and transporters such as allopurinol, bromfenac, and probenecid can manage the blood UA level below the risk threshold, these agents have little benefit for organ health and are commonly linked to side effects, such as gastrointestinal issues and kidney toxicity (Lee et al. 2021).

Traditional Chinese medicine (TCM) classifies HUA under “Bi syndrome” and “Dampness–Turbidity,” attributing its etiology to internal damp‐heat accumulation, phlegm–blood stasis, and spleen–kidney deficiency. According to TCM theory, the spleen governs transportation and transformation. This means that herbs that regulate spleen function are believed to improve metabolic homeostasis. Several Chinese herbs have been reported to exert uric acid–lowering effects, such as Plantaginis Semen (Ou et al. 2026), Lycii Fructus (Yang et al. 2025), and Alismatis Rhizoma (Cheng et al. 2019), etc. Among the various Chinese herbs known for their spleen‐invigorating effects, *Crataegus pinnatifida* Bunge (hawthorn) is one of the earliest recognized medicinal foods, historically used for both culinary and therapeutic purposes (Zhang, Sun, et al. 2022; Zhang, Cui, et al. 2022). It has been widely used since its first documentation in “Xin Xiu Ben Cao” (Bai et al. 2024). While traditionally valued for improving digestion, cardio protection, and lipid reduction, hawthorn has attracted increasing pharmacological interest (Li, Gao, et al. 2023). Modern studies reveal that it is rich in diverse flavonoid compounds (Jurikova et al. 2012), and growing evidence suggests that flavonoids can modulate uric acid metabolism through multiple pathways. For instance, Dihydromyricetin (DMY) and isorhamnetin have been shown to effectively suppress UA production by inhibiting xanthine oxidase (XOD) activity (Kong et al. 2025; Sun et al. 2025). In addition, several flavonoids such as Baicalein (BAL) regulate the expression of renal urate transporters, thereby altering UA reabsorption and secretion to facilitate excretion (Chen et al. 2021; Meng et al. 2017). These findings support the notion that flavonoids exert multi‐target hypouricemic effects (Hu et al. 2009; Wang et al. 2010). As one of the main flavonoids in hawthorn, vitexin (apigenin‐8‐C‐*β*‐D‐glucopyranoside) has gained considerable attention due to its notable efficacy and favorable safety profile in HUA management (He et al. 2016). Accumulating pharmacological evidence indicates that vitexin exhibits multiple bioactivities, including antioxidant effects, regulation of cell proliferation and energy metabolism, and lipid‐lowering properties, highlighting its potential as a natural therapeutic candidate for hyperuricemia (Babaei et al. 2020).

This study began with the extraction and UPLC‐MS/MS‐based identification of flavonoids from hawthorn. Network pharmacology analysis predicted the bioactivities and targets of these flavonoids, highlighting vitexin as the most potent anti‐HUA candidate, with the PPARγ/ABCG2 pathway implicated as its main mechanism. The therapeutic potential of vitexin was then experimentally confirmed in a mouse model of HUA, where biochemical and molecular analyses of major urate transporters consistently validated the network pharmacology predictions.

## 2 Materials and Methods

### 2.1 Materials and Reagents

Vitexin (98%) was purchased from Shaanxi Jinkangtai Biotechnology Co. Ltd. Uricase‐inhibition feed was purchased from Xiaoshu Youtai Co. LTD. (Beijing, China). Benzbromarone was obtained from Aladdin Biotechnology Co. LTD. (Shanghai, China). Kits for determining UA (C012‐2‐1), malondialdehyde (MDA) (A003‐1‐2), ALT (C009‐2‐1) and AST (C010‐2‐1) were purchased from Nanjing Jiecheng Biotechnology Co. LTD. (Nanjing, China); Urea nitrogen test kit (D799850‐0100) was obtained from Sunon Biotechnology Co. LTD. (Beijing, China). Interleukin‐6 (EMC004.96) and Interleukin‐1*β* (EMC104.96) ELISA kits were obtained from Xinbosheng Biotechnology Co. LTD (Shenzhen, China). Antibodies against OAT1 (YP‐Ab‐10825), ABCG2 (YP‐Ab‐00659), Occludin (YP‐Ab‐07715), and ZO‐1 (YP‐Ab‐06280) were obtained from Youpin Biotechnology Co. LTD. (Shenzhen, China). Antibodies against GAPDH (AF7021) and secondary antibody (S0001) were purchased from Jiangsu Kinke Biological Research Center Co. LTD (Jiangsu, China).

### 2.2 Plant Material and Extraction

Hawthorn ( *C. pinnatifida* ) was purchased from Cangxian County, Cangzhou City, Hebei Province, China. The dried plant material was ground and sieved through a 60‐mesh screen. Then, 5 g of the powder was reflux‐extracted twice with 100 mL of 70% ethanol, each for 1 h, in triplicate. The combined extracts were filtered and concentrated under reduced pressure. The concentrate was subjected to purification on a D101 macroporous resin column. After loading for 12 h, the column was rinsed with distilled water and then eluted with 95% ethanol. The ethanol‐eluted fraction was collected and concentrated to obtain the total flavonoid extract of hawthorn (TFH) (Liu et al. 2020). The dried extract was accurately weighed, dissolved in methanol, and filtered through a 0.45 μm membrane prior to mass spectrometric analysis.

### 2.3 UPLC‐MS/MS Analysis of Flavonoids in TFH

Qualitative analysis was conducted using an AB SCIEX Zeno TOF 7600 mass spectrometer coupled with an ultra‐high‐performance liquid chromatography (UPLC) system in both positive and negative electrospray ionization (ESI) modes. Data acquisition employed an information‐dependent acquisition (IDA) strategy, enabling automated switching between full‐scan and targeted MS/MS modes (Decaestecker et al. 2000).

Chromatographic separation was achieved using an Acquity UPLC BEH C18 column (2.1 × 100 mm, 1.8 μm; Waters, USA) maintained at 40°C. The mobile phase consisted of (A) water and (B) acetonitrile, delivered at a flow rate of 0.3 mL·min^−1^. An injection volume of 3 μL was used for each sample (Huang et al. 2014).

The mass spectrometry parameters were optimized according to the manufacturer's recommendations for small molecule analysis on the Zeno TOF 7600 system. The optimized parameters were as follows: full‐scan and MS/MS spectra were acquired over an m/z range of 50–1500. Nebulizing and heating gas pressures were set to 55 psi, with a curtain gas pressure of 35 psi. The ion source temperature was 550°C, and the ion spray voltage was maintained at +5.5 kV. Additional parameters included a declustering potential of 60 V, collision energy of 10 V, and a cone voltage of 35 ± 15 V. The accumulation time was 0.25 s per acquisition cycle.

### 2.4 Network Pharmacology Analysis

#### 2.4.1 Construction of a Flavonoid Database From TFH

**FIGURE 1:**
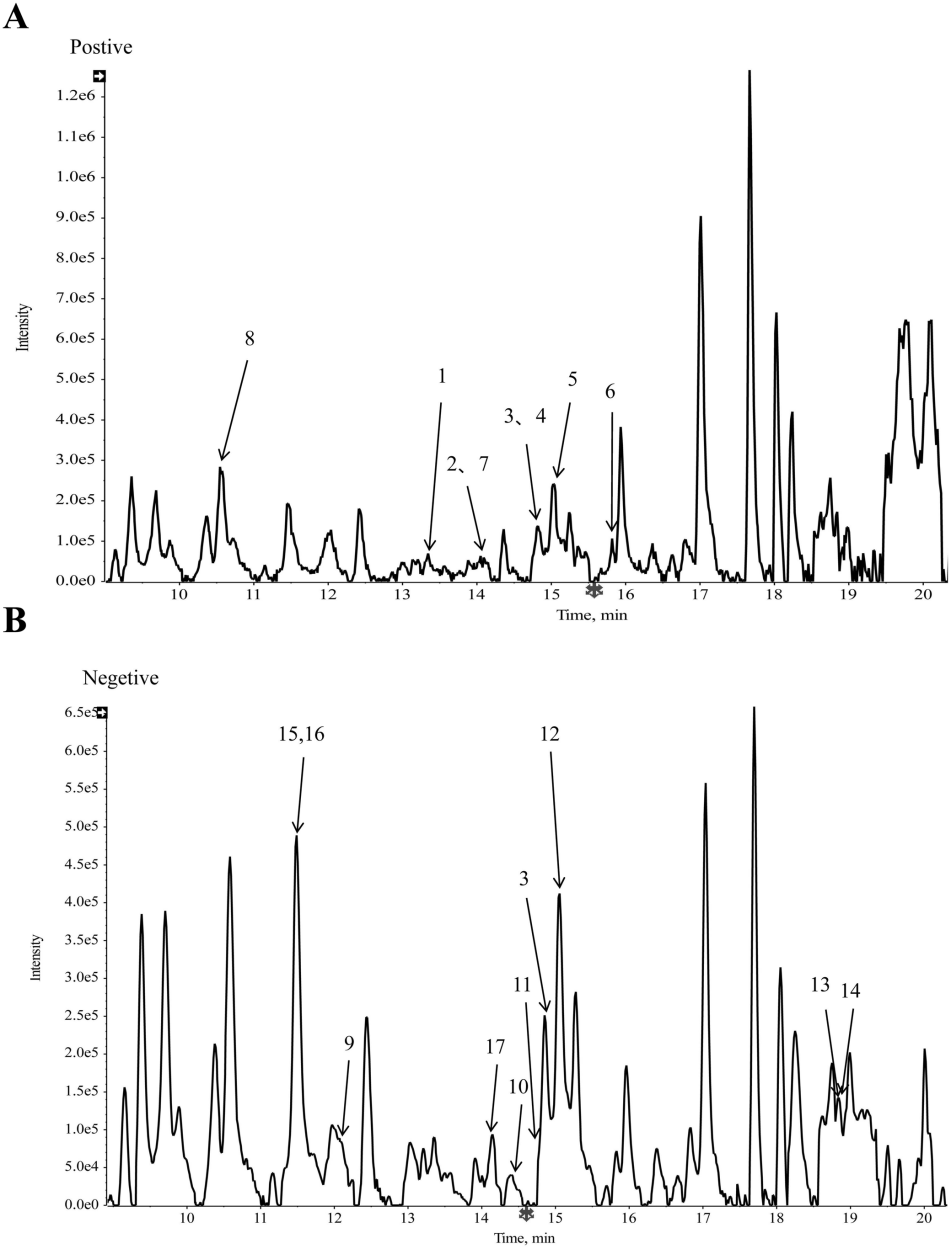
Overlaid total ion chromatograms (TICs) of TFH obtained from LC–MS analysis. (A) Positive electrospray ionization (ESI^+^) mode. (B) Negative electrospray ionization (ESI^−^) mode.

The raw LC–MS data were processed using AB SCIEX PeakView software, and the resulting total ion chromatogram (TIC) is presented in Figure 1. The acquired data were converted using MSconvert and further analyzed using SIRIUS software to elucidate ion structures and identify flavonoid compounds present in the crude TFH extract, supported by literature evidence (Cui et al. 2024) and database matching. Quantification of the detected flavonoids was performed in MZmine based on peak area analysis. All identified compounds were cross‐referenced against the PubChem database to obtain their corresponding SMILES notations. Potential molecular targets and pharmacokinetic properties were then predicted using the SwissADME platform. Based on these results, a customized flavonoid database was constructed to support subsequent network pharmacology prediction (Wu et al. 2024).

#### 2.4.2 Prediction of Potential Therapeutic Targets

SMILES notations of the identified flavonoids were retrieved from PubChem and submitted to the SwissTargetPrediction server, with *Homo sapiens* selected as the target species (Daina et al. 2019). Concurrently, hyperuricemia (HUA)‐related targets were collected from the GeneCards and OMIM databases using “hyperuricemia” as the search term. Potential therapeutic targets were identified by intersecting the predicted compound targets with the HUA‐associated gene sets (Amberger et al. 2019).

#### 2.4.3 Identification of Common Targets for HUA and TFH

An online Venn diagram tool was used to identify the overlapping targets between TFH‐derived flavonoids and HUA‐related genes, establishing a set of intersecting genes for subsequent analysis (Tang et al. 2023).

#### 2.4.4 Protein–Protein Interaction (PPI) Network Construction

The common targets were imported into the STRING database to construct a PPI network, restricted to *Homo sapiens* (Xu et al. 2019). The resulting network was imported into Cytoscape (v3.7.1/v3.9.1) for topological analysis. Key hub targets were identified based on network centrality parameters, including degree, closeness, and betweenness.

#### 2.4.5 GO and KEGG Enrichment Analysis

Functional enrichment analysis of the intersecting genes was performed using the DAVID database. Gene Ontology (GO) enrichment covered biological process (BP), cellular component (CC), and molecular function (MF) categories. Kyoto Encyclopedia of Genes and Genomes (KEGG) pathway enrichment was also conducted to identify relevant biological pathways (Zou et al. 2025).

### 2.5 Molecular Docking and Molecular Dynamics Simulations

The crystal structures of PPARγ (PDB: 3DZY) and ABCG2 (PDB: 6ETI) were obtained from the RCSB Protein Data Bank. The 3D structure of vitexin was constructed in ChemBio3D 22.2.0, energy‐minimized, and saved in .mol2 format. Protein structures were prepared using AutoDock Tools 1.5.7, which included removing water molecules and adding polar hydrogens, following established protocols (Jakhar et al. 2020). Molecular docking was performed using the prepared proteins and ligands, with binding affinities and interaction modes analyzed and visualized in PyMOL.

To evaluate the stability of the docking complexes, all‐atom molecular dynamics (MD) simulations were performed using GROMACS 2024.03 (Abraham et al. 2015). The initial PPARγ–vitexin complex was derived from the optimal docking pose. Protein parameters were assigned using the Amber14SB force field, and ligand topologies were generated with ACPYPE based on the GAFF force field (Maier et al. 2015). The system was solvated in a TIP3P water box under periodic boundary conditions and neutralized with Na^+^/Cl^−^ ions at a physiological concentration of 0.15 mol/L (Jorgensen et al. 1983). The system underwent energy minimization via the steepest descent algorithm, followed by NVT and NPT equilibration (100 ps each) with positional restraints on protein heavy atoms. Production MD was run for 100 ns with a 2‐fs time step. Long‐range electrostatic interactions were treated using the particle mesh Ewald (PME) method, and trajectories were saved every 10 ps for analysis.

Trajectory analyses included root‐mean‐square deviation (RMSD), root‐mean‐square fluctuation (RMSF), radius of gyration (Rg), hydrogen bonding, principal component analysis (PCA), free‐energy landscape (FEL) mapping, and Ramachandran assessment. The binding free energy (ΔG_bind) was calculated using the MM/GBSA method with gmx_MMPBSA, decomposing contributions from van der Waals, electrostatic, polar solvation, and non‐polar solvation terms (Valdés‐Tresanco et al. 2021). These analyses provided a comprehensive evaluation of the dynamic behavior and binding stability of the PPARγ–vitexin complex.

### 2.6 Animal Experiment Design

Male C57BL/6J mice (8 weeks old, 20 ± 2 g, specific pathogen‐free) were purchased from Zhuhai BesTest Biotechnology Co. Ltd. (Zhuhai, China). Animals were housed under SPF conditions at the Jiangmen International Institute of Health Innovation, in compliance with Chinese national standards for laboratory animal environments. All mice were maintained on a 12 h light/dark cycle with free access to standard diet and water. After a 1‐week acclimation period, mice were randomly assigned to a normal control group, a hyperuricemia (HUA) model group, a vitexin treating group, and a positive control group, with nine mice in each group. The experimental design and treatment schedule are illustrated in Figure 4A.

The HUA model was induced by feeding a diet containing 0.2% adenine and 5% potassium oxonate (PO) for 14 days, as described in previous studies (Li, Luan, et al. 2023) while the control group continued on a normal diet. After modeling, blood was collected from the retro‐orbital plexus under isoflurane anesthesia to confirm elevated plasma uric acid (UA) levels. Plasma UA levels were subsequently monitored on a weekly basis during the treatment phase. Although UA levels were not assessed during the early period following PO discontinuation, they remained significantly higher in the model group than in controls throughout the intervention period (Figure 4C,D), indicating sustained elevation of UA during the treatment window. The same blood collection procedure was performed in the normal control group to maintain consistency across groups.

HUA mice were then divided into treatment subgroups: a positive control group receiving 40 mg/kg benzbromarone, a vitexin‐treated group administered 100 mg/kg vitexin, and a model control group given 0.5% CMC–Na solution (Li, Gao, et al. 2023; Li et al. 2025). The normal control group also received vehicle. All treatments were delivered once daily by oral gavage for 21 consecutive days.

### 2.7 Sample Collection and Processing

After a 12 h fast on the final day of treatment, blood was collected from anesthetized mice, which were then euthanized. The liver, kidneys, ileum, and colon were rapidly excised. Portions of each tissue were fixed in 4% paraformaldehyde for 24 h and embedded in paraffin for histology. The remaining tissues were snap‐frozen in liquid nitrogen and stored at −80°C. Blood samples were kept at 4°C for 2 h, centrifuged at 3500 rpm for 20 min at 4°C, and the resulting plasma was stored at −80°C for subsequent biochemical analysis, as previously described (Li et al. 2025).

### 2.8 Biochemical Analysis

Plasma levels of UA, ADA, XOD, CREA, UREA, ALT, AST, MDA, IL‐6, and IL‐1β, as well as hepatic XOD activity, were measured using commercial assay kits according to the manufacturers' instructions.

### 2.9 Histopathological Examination

Formalin‐fixed liver, kidney, colon, and ileum tissues were embedded in paraffin, sectioned at 4 μm, and stained with hematoxylin and eosin (H&E). Histopathological changes were evaluated under bright‐field microscopy (Olympus, Japan) at 200× magnification.

### 2.10 Gene Expression

Total RNA was extracted from kidney and colon tissues using commercial kits and reverse‐transcribed into cDNA. Quantitative PCR was performed using SYBR Green reagents on a CFX96 Detection System (Bio‐Rad, USA). All primers were synthesized by Sangon Biotech (Shanghai, China). Gene expression was normalized to a reference gene and analyzed using the 2^−ΔΔCT^ method (Bustin 2024; Schmittgen 2001).

### 2.11 Immunoblotting Analysis

Renal and colonic proteins were extracted using RIPA lysis buffer (Biosharp, China). Protein concentrations were determined by BCA assay, and equal amounts of protein were separated by SDS–PAGE and transferred to PVDF membranes (Millipore, USA) (Wang and Wang 2025). After blocking with 5% non‐fat milk, membranes were incubated overnight at 4°C with primary antibodies against GAPDH, OAT1, ABCG2, ZO‐1, and occludin (Upingbio, China), followed by incubation with HRP‐conjugated secondary antibodies (Taylor et al. 2019). Protein bands were visualized using an ECL detection kit (Biosharp, China).

### 2.12 Statistical Analysis

Data are presented as mean ± SEM and were analyzed using GraphPad Prism. After verifying normality and homogeneity of variance, one‐way ANOVA with LSD post hoc test was applied for multiple comparisons; non‐parametric tests were used when assumptions were not met. A *p*‐value < 0.05 was considered statistically significant.

## 3 Results

### 3.1 Identification of Flavonoids in TFH Through LC–MS Analysis

LC–MS analysis was performed to characterize the flavonoid composition of TFH (Figure 1). Mass spectrometry data were acquired in both positive and negative ionization modes on an AB SCIEX Zeno TOF 7600 system and processed using Analyst TF 1.7. A total of 17 flavonoids were identified by matching with literature reports (Cui et al. 2024) and database annotations via the SIRIUS platform (v5.8.3). Detailed chemical information—including retention time, molecular formula, exact mass, and peak area—is provided in Table 1. After determining that spiraeoside, hyperoside, and vitexin were the most abundant compounds, their drug‐like properties were profiled using SwissADME (Supporting Information). This analysis identified vitexin, with its decent drug‐like characteristics and promising oral bioavailability (0.55 vs. 0.17 for spiraeoside and hyperoside), as the prime candidate for further mechanistic and pharmacodynamic validation.

**TABLE 1:** Identification of the main flavonoids in TFH by LC–MS/MS.

Compounds	RT (min)	Charge state	Error (ppm)	Measured (m/z)	Predicted (m/z)	Formula	Name	Peak area
1	13.34	[M + H]	−1.833	757.2189	757.2113	C_33_H_40_O_20_	Quercetin‐3‐*O*‐[2,6‐di‐*α*‐L‐rhamnopyranosyl‐*β*‐D‐galactopyranoside]	100,000
2	14.12	[M + H]	−1.083	611.1621	611.1534	C_27_H_30_O_16_	Rutin	75,000
3	14.82	[M + H]	−1.876	433.1129	433.1056	C_21_H_20_O_10_	Vitexin	180,000
4	14.82	[M + Na]	2.803	455.0950	455.1056	C_21_H_20_O_10_	Isovitexin	60,000
5	15.03	[M + H]	−0.197	465.1027	465.0955	C_21_H_20_O_12_	Hyperoside	220,000
6	15.67	[M + H]	−5.690	389.1209	389.1158	C_20_H_20_O_8_	5‐Hydroxyauranetin	8100
7	10.56	[M + H]	−0.778	867.2125	867.2058	C_45_H_38_O_18_	Procyanidin C1	5000
8	14.12	[M + H]	0.818	741.183	741.1741	C_39_H_32_O_15_	Kandelin A1	75,000
9	12.18	[M − H]	−4.607	593.1494	593.1585	C_27_H_30_O_15_	Kaempferol 3‐neohesperidoside	9000
10	14.46	[M − H]	−2.898	593.1494	593.1585	C_27_H_30_O_15_	Vitexin‐4″‐*O*‐*β*‐D‐glucopyranoside	49,000
11	14.78	[M − H]	−6.06	577.1539	577.1636	C_27_H_30_O_14_	Vitexin‐2″‐*O*‐*α*‐L‐rhamnopyranoside	29,000
12	15.04	[M − H]	−1.295	463.0876	463.0955	C_21_H_20_O_12_	Spiraeoside	410,000
13	18.80	[M − H]	−6.271	285.0401	285.0477	C_15_H_10_O_6_	Luteolin	10,000
14	18.81	[M − H]	−2.642	301.0348	301.0427	C_15_H_10_O_7_	Quercetin	51,000
15	11.49	[M − H]	−5.387	303.0511	303.0583	C_15_H_12_O_7_	(+)‐Taxifolin	40,000
16	11.49	[M + Cl]	−3.028	325.0473	325.079	C_15_H_14_O_6_	Catechin	33,000
17	14.09	[M − H]	−2.933	577.1334	577.1424	C_30_H_26_O_12_	Procyanidin B2	49,000

### 3.2 Network Pharmacology Analysis of TFH

**FIGURE 2:**
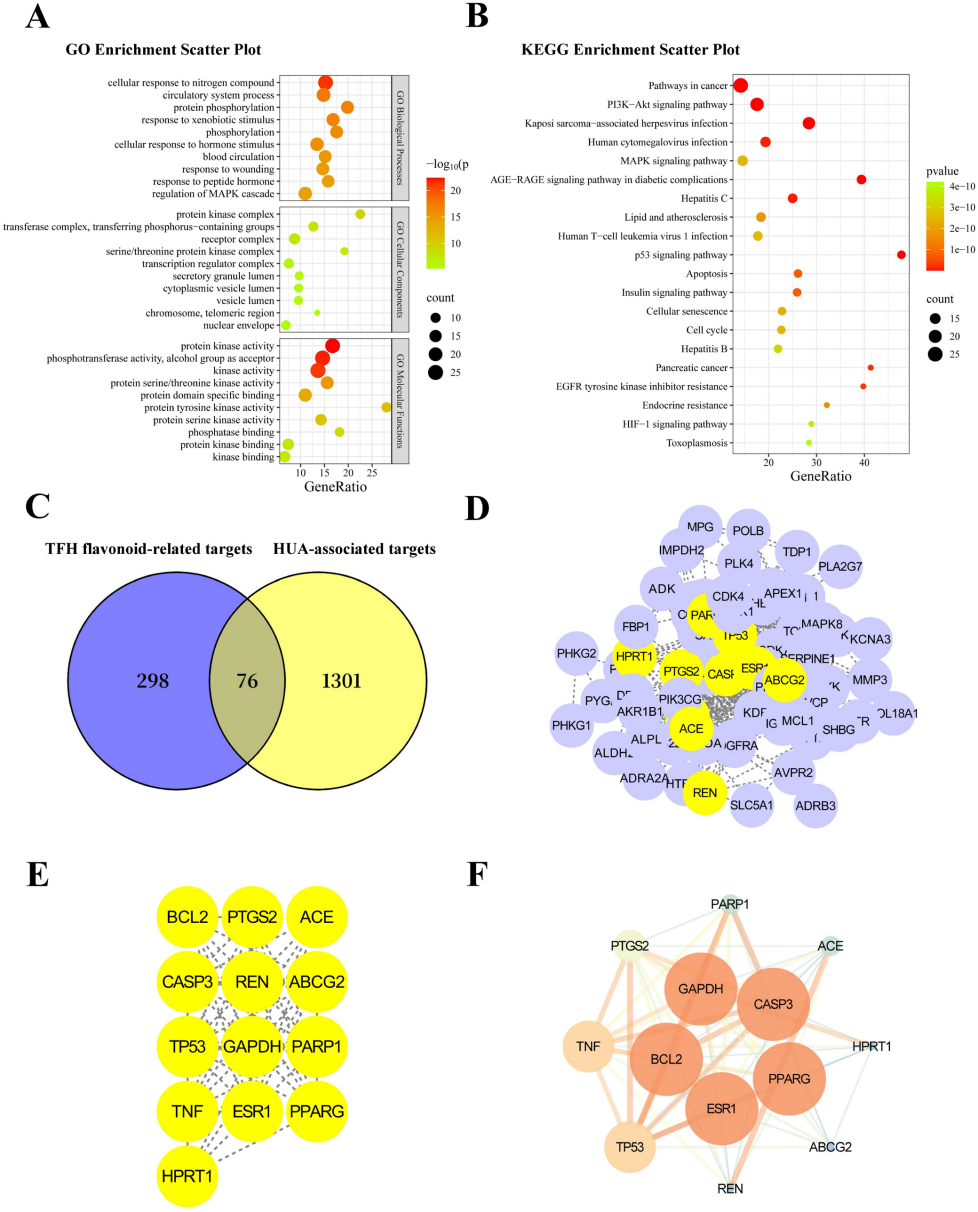
Integrated network pharmacology analysis of TFH against hyperuricemia. (A) Gene ontology (GO) enrichment analysis of biological processes (BP), cellular components (CC), and molecular functions (MF) conducted using the ClusterProfiler R package. (B) Kyoto encyclopedia of genes and genomes (KEGG) pathway enrichment analysis of the predicted targets. (C) Venn diagram showing the overlap between TFH flavonoid‐related targets (298) and HUA‐associated targets (1301), with 76 overlapping targets considered potential therapeutic targets. (D) TFH–disease target interaction network. (E) Protein–protein interaction (PPI) network of the key targets. (F) Refined core PPI network highlighting the major hub genes.

Network pharmacology analysis predicted 374 potential targets for these flavonoids and 1375 targets associated with hyperuricemia (HUA), with 76 overlapping targets considered potentially relevant to TFH's anti‐HUA effects (Figure 2C,D).

A protein–protein interaction (PPI) network was constructed using the STRING database and analyzed in Cytoscape. Topological analysis identified 13 hub targets with high connectivity, including PPARγ, CASP3, BCL2, ESR1, ABCG2, etc. (Figure 2E,F). Among these hub targets, PPARγ/ABCG2 were prioritized for further investigation based on their reported roles in urate transport regulation (Han et al. 2025; Xu et al. 2024). Functional enrichment analysis also clarified the biological relevance of the overlapping targets. Gene Ontology (GO) analysis indicated that the top 10 core genes were enriched in processes such as positive regulation of the MAPK cascade, response to xenobiotic stimuli, and protein phosphorylation (Figure 2A). KEGG pathway analysis suggested the PI3K‐Akt signaling pathway as significantly enriched (Figure 2B). These enrichment results described the functional categories and potential signaling pathways associated with the predicted target set.

### 3.3 Molecular Docking and Molecular Dynamics Study

**FIGURE 3:**
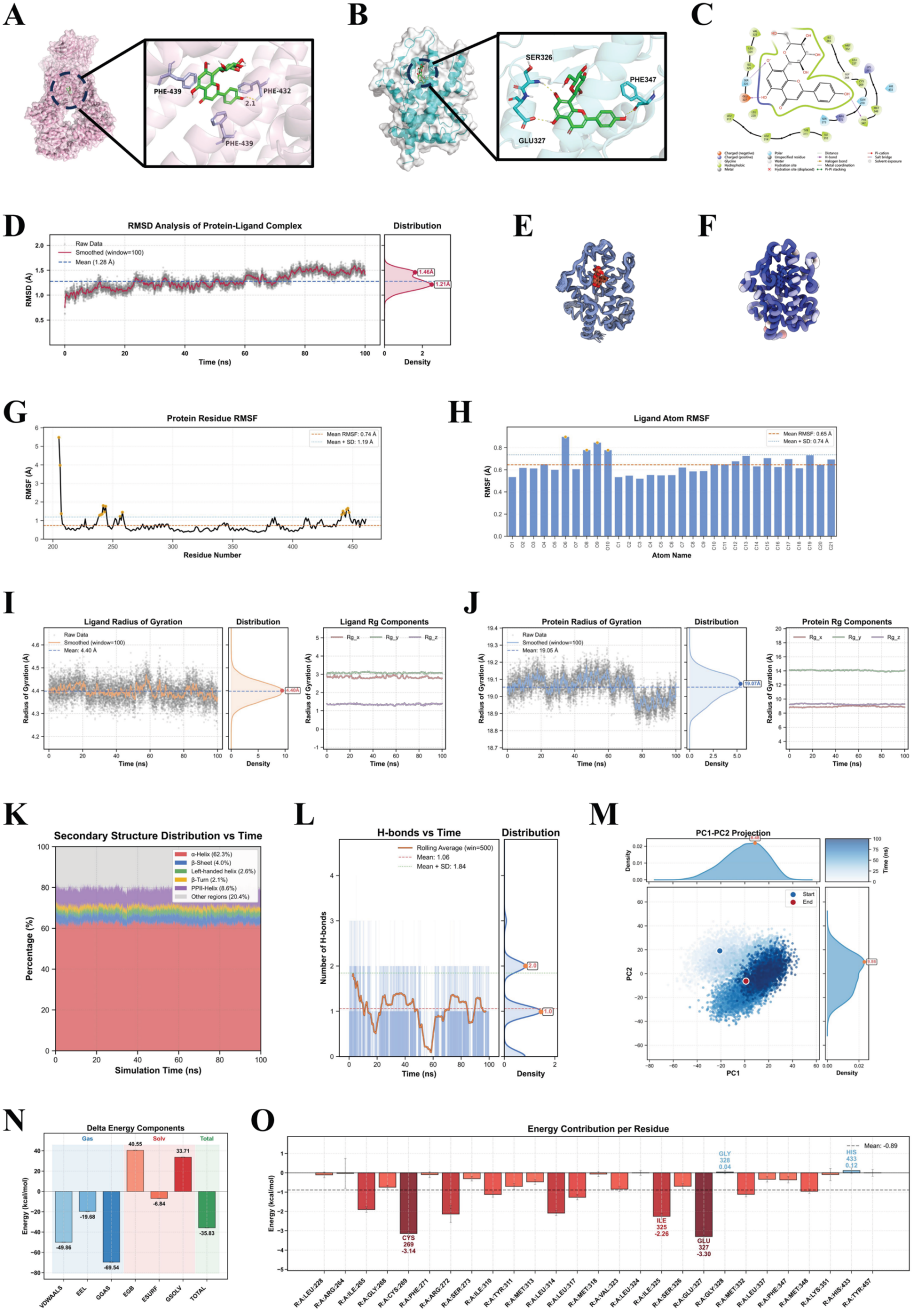
Molecular docking and molecular dynamics (MD) analyses of the ABCG2–vitexin and PPARγ–vitexin complexes. (A) Three‐dimensional binding pose of vitexin within the ABCG2 binding site, with an enlarged view of the interaction environment. (B) Binding pose of vitexin in the PPARγ pocket and the corresponding enlarged interaction view. (C) Two‐dimensional interaction diagram showing key hydrogen‐bond and hydrophobic contacts. (D) RMSD evolution of the PPARγ–vitexin complex. (E) Superimposition of the final 100 snapshots from the MD trajectory. (F) B‐factor analysis revealing residue mobility. (G) RMSF profile of PPARγ residues. (H) RMSF of vitexin atoms. (I) Time‐dependent radius of gyration (*Rg*) of PPARγ and its principal components. (J) *Rg* variation of vitexin. (K) Secondary‐structure evolution of PPARγ. (L) Number of hydrogen bonds formed between vitexin and PPARγ. (M) PCA distribution of MD conformations along PC1 and PC2. (N) Binding free energy obtained from MM/GBSA analysis. (O) Per‐residue energy decomposition showing key residues contributing to ligand binding.

As vitexin was established as the primary bioactive component of TFH and PPARγ/ABCG2 were predicted as potential targets for HUA management, their interactions were further investigated using structure‐based approaches. Consistent with network pharmacology predictions, vitexin demonstrated promising binding affinities toward both PPARγ and ABCG2, with docking energies of −5.66 and −7.83 kcal/mol, respectively. These free energy (ΔG) values suggest spontaneous binding of vitexin to the ligand‐binding domains of both proteins. A detailed interaction analysis further revealed that vitexin forms a network of stabilizing contacts, involving hydrogen bonds with key residues such as PHE432, PHE439, PHE347, SER326, and GLU327, in addition to hydrophobic interactions and π–π stacking. This multifaceted binding profile provides a structural basis for the potential of vitexin to interact with urate‐related regulatory pathways via ABCG2 (Figure 3A) and PPARγ (Figure 3B) (Yan et al. 2025).

Given PPARγ's higher centrality in the network pharmacology analysis (Figure 2F), its established role as an upstream regulator of ABCG2, and its well‐characterized ligand‐binding domain, it was prioritized for further MD simulation to explore binding dynamics. To benchmark the docking results, MRL24 (a known PPARγ agonist) was included as a positive control (Choi et al. 2011), which showed strong binding (−10.55 kcal/mol) and a similar binding orientation to vitexin (Supporting Information). To validate the predicted binding pose and explore the dynamic behavior of vitexin within the PPARγ binding pocket, a 100‐ns all‐atom molecular dynamics (MD) simulation was conducted using GROMACS 2024.03, with the Amber14SB force field applied to the protein and GAFF parameters to the ligand. The initial binding conformation of vitexin, derived from AlphaFold3 predictions, showed hydrogen‐bond interactions with residues SER326, GLU327, and PHE347, alongside hydrophobic contacts with LEU314 (Figure 3B,C) (Abramson et al. 2024).

Trajectory analysis indicated that the backbone root‐mean‐square deviation (RMSD) of PPARγ rapidly stabilized within the initial nanoseconds and remained within a narrow range throughout the simulation, averaging 1.28 Å (Figure 3D). Vitexin itself exhibited similarly restrained RMSD fluctuations, with a mean value of 0.50 Å, reflecting its stable positioning within the binding site. Structural superposition of the final 10 ns (100 frames) further suggested the conformational consistency of the PPARγ–vitexin complex (Figure 3E) (Yang et al. 2021).

B‐factor analysis of residue‐level mobility indicated limited fluctuations within the ligand‐binding cavity, whereas greater flexibility was observed in the N‐terminal and loop regions (Figure 3F). Root‐mean‐square fluctuation (RMSF) profiles indicated motions for most PPARγ residues, with an average RMSF of 0.74 Å (Figure 3G), while RMSF of vitexin atoms averaging 0.65 Å (Figure 3H) (Agosta and Cozzini 2024). The radius of gyration (Rg) of PPARγ remained largely invariant, averaging 19.05 Å, and its principal axes showed minimal variation (Figure 3I). Corresponding Rg values for vitexin and its axial components averaged 4.40 Å (Figure 3J), reflecting both protein compactness and ligand structural stability (Azam and Bello 2022).

Secondary‐structure analysis indicated that PPARγ largely preserved its native fold during the simulation, with α‐helices accounting for 62.3%, β‐sheets for 4.0%, and other structural elements remaining largely unchanged (Figure 3K). Hydrogen‐bond monitoring demonstrated that one to three persistent interactions were maintained between Vitexin and residues GLU327 and SER326, averaging 1.06 hydrogen bonds over the simulation period (Figure 3L).

Principal component analysis revealed a progressive convergence of the system into a confined conformational space, with the first two components capturing the majority of structural variations (Figure 3M). The free‐energy landscape indicated a dominant low‐energy state, supporting the overall conformational stability of the complex. MM/GBSA calculations yielded a mean binding free energy of −35.83 kcal/mol, with van der Waals (−49.86 kcal/mol) and electrostatic (−19.68 kcal/mol) interactions contributing most prominently (Figure 3N). Per‐residue energy decomposition identified GLU327, ILE325, and CYS269 as key contributors to ligand binding (Figure 3O).

### 3.4 Vitexin Alleviates Hyperuricemia in a Mouse Model Induced by Adenine and Potassium Oxonate

**FIGURE 4:**
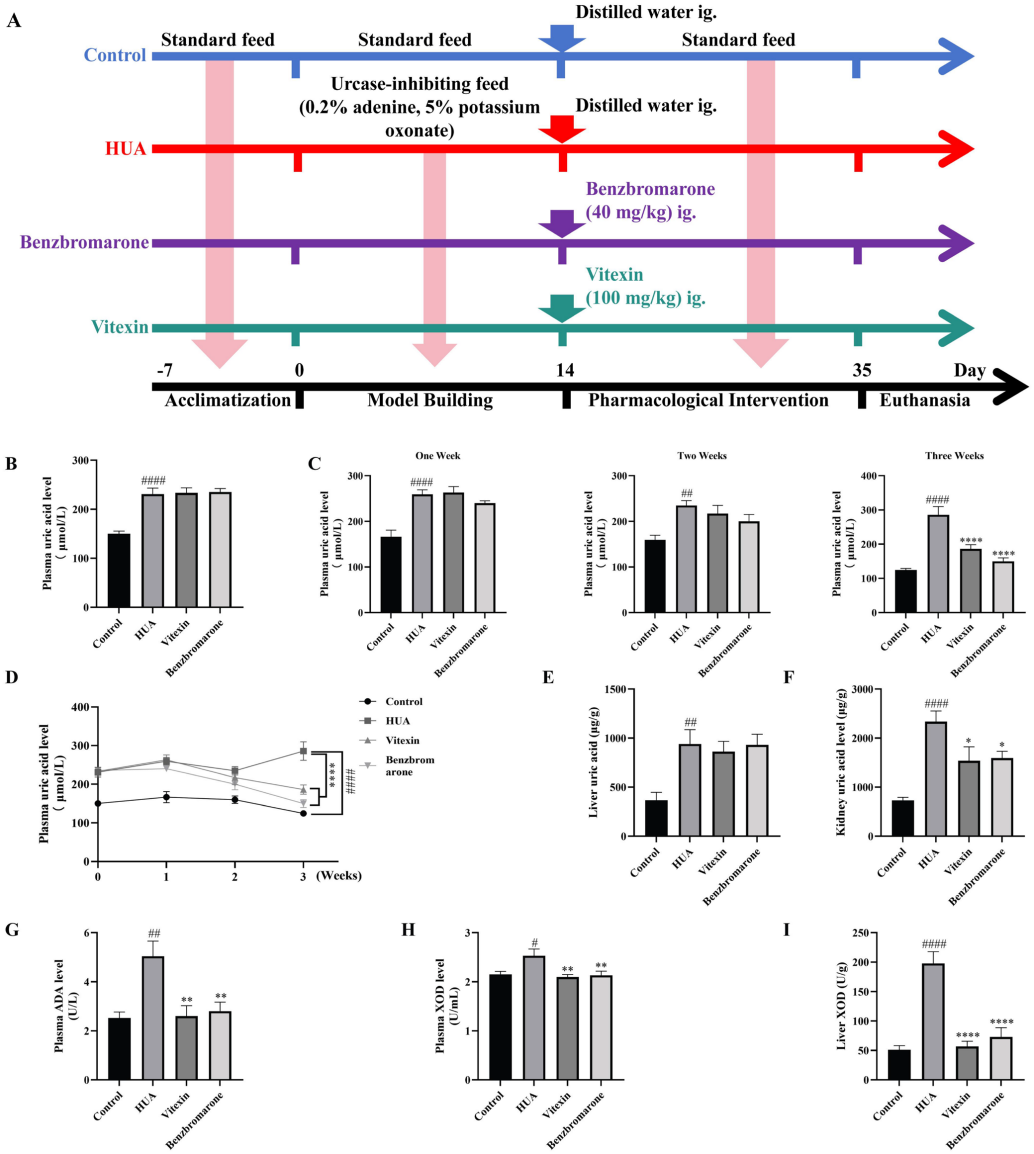
The therapeutic effects of vitexin (100 mg/kg) and benzbromarone (40 mg/kg) in HUA mice. (A) Schematic diagram of the animal experiments. (B) Plasma UA levels in mice after the modeling procedure (*n* = 8–9). (C) Plasma UA levels after 1, 2, and 3 weeks of treatment (*n* = 6–9). (D) Alterations in plasma UA levels in mice during treatment (*n* = 6–9). (E) UA levels in liver tissue (*n* = 6). (F) UA levels in kidney tissue (*n* = 5–7). (G) ADA activity in plasma (*n* = 6–8). (H) XOD activity in plasma (*n* = 5–7). (I) XOD activity in liver (*n* = 6–8). Data are represented as mean ± SEM. ^#/*^ *p* < 0.05, ^##/**^ *p* < 0.01, ^###/***^ *p* < 0.001, ^####/****^ *p* < 0.0001, ^#^vs. Control group, *vs. HUA group.

An adenine and potassium oxonate‐induced hyperuricemic mouse model was employed to experimentally validate the regulatory effect of vitexin on uric acid metabolism (Figure 4A). Plasma UA level is a key diagnostic and evaluative biomarker for HUA. As shown in Figure 4B, mice treated with 0.2% adenosine and 5% sodium oxonate exhibited a marked increase in plasma UA compared to the control group (*p* < 0.0001). Notably, after 3 weeks of vitexin administration, plasma UA levels were significantly reduced relative to the HUA model group (*p* < 0.001, Figure 4C,D). Since UA is predominantly produced in the liver and excreted via the kidneys, tissue‐specific UA accumulation was further assessed. Vitexin showed a trend toward reduction in hepatic tissue (Figure 4E) while significantly decreasing renal UA content (*p* < 0.05; Figure 4F), comparable to those of benzbromarone. The adenosine‐potassium oxonate model significantly elevated plasma ADA activity (*p* < 0.01; Figure 4G) and increased XOD activity in both plasma and liver (*p* < 0.05 and *p* < 0.0001, respectively; Figure 4H,I), indicating enhanced purine catabolism and pathological UA accumulation.

### 3.5 Vitexin Promotes UA Excretion by Regulating UA Transporter Expression

**FIGURE 5:**
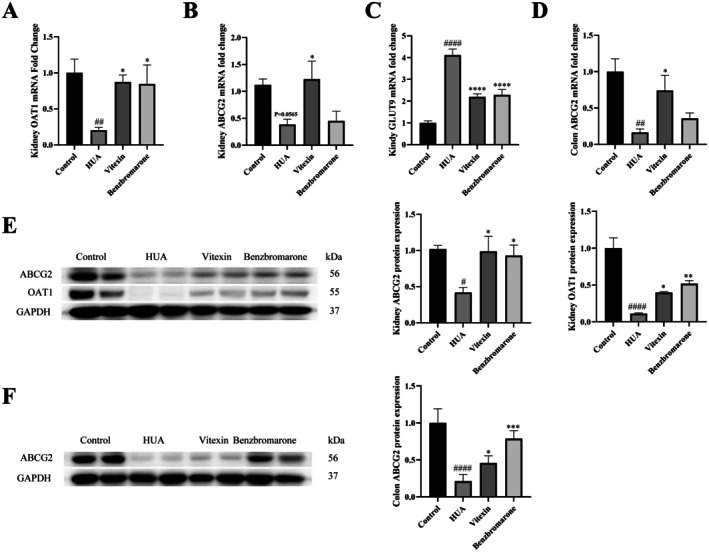
Effects of vitexin on the expression of uric acid transporters in kidney and colon tissue of hyperuricemic mice. (A) Gene expression levels of OAT1 in mouse kidney tissue (*n* = 6). (B) Gene expression levels of ABCG2 in mouse kidney tissue (*n* = 6). (C) Gene expression levels of GLUT9 in mouse kidney tissue (*n* = 6–7). (D) Gene expression levels of ABCG2 in mouse colon tissue (*n* = 5–6). (E) Protein expression levels of ABCG2 and OAT1 in mouse kidney tissue (*n* = 4). (F) Protein expression level of ABCG2 in mouse colon tissue (*n* = 4). Data are represented as mean ± SEM. ^#/*^ *p* < 0.05, ^##/**^ *p* < 0.01, ^###/***^ *p* < 0.001, ^####/****^ *p* < 0.0001, ^#^vs. Control group, *vs. HUA group.

To elucidate the molecular mechanisms underlying vitexin's regulatory effect on UA transport, the expression of these transporters in renal and colonic tissues was analyzed using qPCR and Western blot. In HUA model mice, the expression pattern of transporters differed substantially from that in the control group. Transcript levels of the renal excretory transporters OAT1 and ABCG2 were largely downregulated (*p* < 0.05, Figure 5A; *p* = 0.0565, Figure 5B), whereas the reabsorptive transporter GLUT9 was markedly upregulated (*p* < 0.0001, Figure 5C). These changes were consistent at the protein level, with ABCG2 and OAT1 expression significantly decreased (*p* < 0.001 or *p* < 0.0001; Figure 5E). In colonic tissues, both mRNA and protein levels of ABCG2 were reduced in the HUA group (Figure 5D,F). Vitexin treatment effectively reversed these molecular alterations. In the kidney, it significantly upregulated ABCG2 and OAT1 mRNA (*p* < 0.05; Figure 5A,B) and downregulated GLUT9 mRNA (*p* < 0.0001; Figure 5C), accompanied by increased protein expression of ABCG2 and OAT1 (Figure 5E). In the colon, vitexin restored the suppressed transcription (*p* < 0.05; Figure 5D) and translation (*p* < 0.001; Figure 5F) of ABCG2.

### 3.6 Vitexin Ameliorates HUA‐Induced Liver and Kidney Injury

**FIGURE 6:**
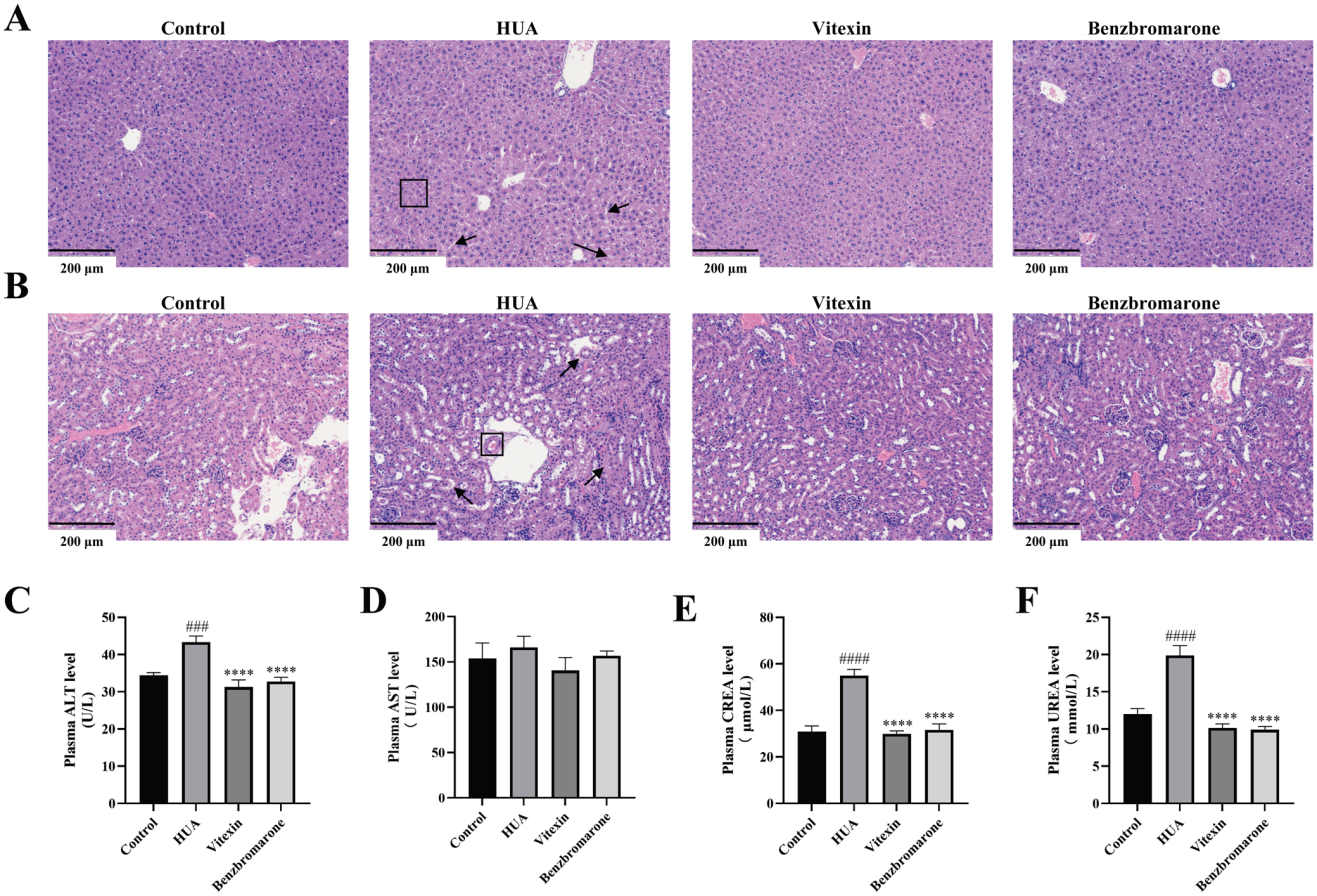
Vitexin (100 mg/kg) treatment alleviates liver and kidney damage in hyperuricemic mice. (A) Representative images of HE staining of liver tissue (200×, scale bar: 200 μm). (B) Representative images of HE staining of kidney tissue (200×, scale bar: 200 μm). (C) Plasma ALT levels (*n* = 5–8). (D) Plasma AST levels (*n* = 5–7). (E) Plasma creatinine levels (*n* = 7–9). (F) Plasma urea nitrogen levels (*n* = 7–9). Data are represented as mean ± SEM. ^####/****^ *p* < 0.0001, ^#^vs. control group, *vs. HUA group.

Histological examination via hematoxylin and eosin (H&E) staining revealed significant hepatic injury in HUA model mice, characterized by extensive edema, ballooning degeneration, hepatocyte swelling with cytoplasmic rarefaction, and vacuolar degeneration (Figure 6A). In renal tissues, HUA induction led to tubular dilation, epithelial cell vacuolization, glomerular atrophy, and mild inflammatory infiltration (Figure 6B). Treatment with either vitexin or benzbromarone markedly attenuated these structural abnormalities.

Consistent with the histopathological findings, biochemical analyses indicated pronounced hepatic and renal impairment in HUA mice. Plasma level of alanine aminotransferase (ALT) was significantly elevated in the HUA group, and aspartate aminotransferase (AST) was relatively increased, and these markers of liver injury were considerably reduced by vitexin administration (Figure 6C,D). On the other hand, renal function parameters—creatinine and urea—were markedly increased in HUA mice relative to controls (*p* < 0.0001; Figure 6E,F), indicating compromised kidney function. Notably, vitexin treatment effectively restored both creatinine and urea concentrations to levels comparable to those achieved with benzbromarone (*p* < 0.0001; Figure 6E,F).

### 3.7 Vitexin Attenuates Intestinal Barrier Damage and Inflammation in HUA Mice

**FIGURE 7:**
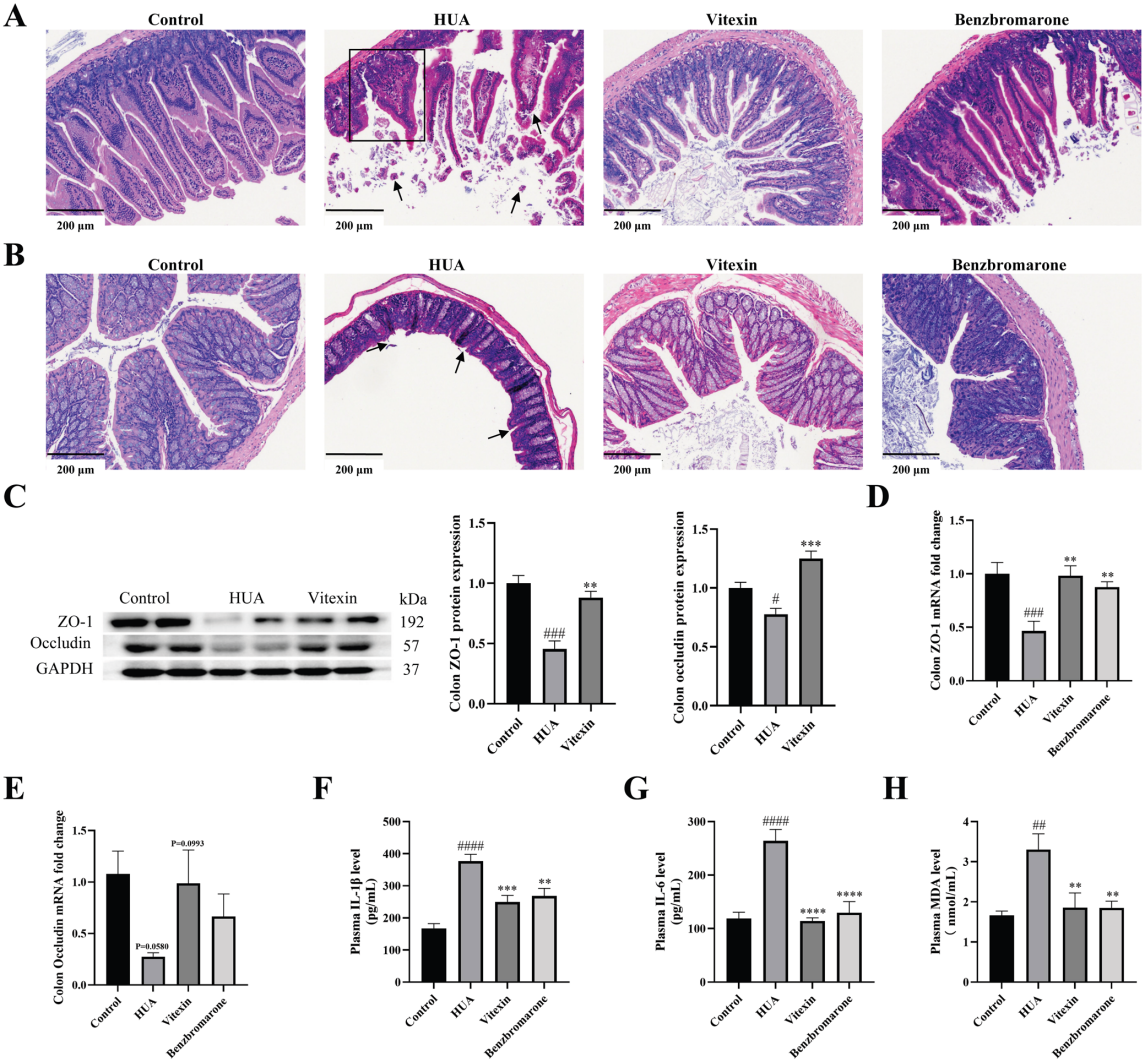
Effects of vitexin on intestinal barrier integrity and inflammation in hyperuricemic mice. (A) Representative images of HE staining of the ileum tissue (200×, scale bar: 200 μm). (B) Representative images of HE staining of the colon tissue (200×, scale bar: 200 μm). (C) Protein expression levels of ZO‐1 and occludin in mouse colon tissue (*n* = 4). (D) Gene expression levels of ZO‐1 in mouse colon tissue. (E) Gene expression levels of occludin in mouse colon tissue. (F) IL‐1β levels in plasma. (G) IL‐6 levels in plasma. (H) MDA levels in plasma. Data are represented as mean ± SEM (*n* = 6 unless otherwise indicated). ^#/*^ *p* < 0.05, ^##/**^ *p* < 0.01, ^###/***^ *p* < 0.001, ^####/****^ *p* < 0.0001, ^#^vs. control group, *vs. HUA group.

The effect of vitexin on intestinal integrity in HUA mice was further investigated. H&E staining of ileal and colonic sections from HUA mice revealed substantial morphological damage, including villous shortening and loss of structural integrity in the ileum (Figure 7A), as well as crypt atrophy, reduced goblet cell numbers, and mucosal degeneration in the colon (Figure 7B). These pathological changes were markedly ameliorated by treatment with vitexin or benzbromarone.

To explore the mechanisms underlying intestinal barrier disruption, the expression of tight junction proteins was assessed by Western blot and qPCR. Compared with controls, HUA mice exhibited significantly reduced protein and mRNA levels of ZO‐1 and occludin in colonic tissues (Figure 7C–E). Vitexin treatment effectively restored the expression of these key tight junction components, indicating a protective effect on intestinal epithelial barrier function.

Furthermore, systemic inflammation and oxidative stress levels were also measured. HUA mice showed significantly elevated plasma levels of the pro‐inflammatory cytokines IL‐6 and IL‐1β (*p* < 0.0001; Figure 7F,G), as well as increased malondialdehyde (MDA), a marker of oxidative stress (*p* < 0.01; Figure 7H). Vitexin treatment significantly suppressed these inflammatory and oxidative mediators, underscoring its dual anti‐inflammatory and antioxidant effects in the context of HUA.

## 4 Discussion

### 4.1 LC–MS Analysis, Computational Prediction, and the Corresponding Experimental Findings

The discovery of safer and more effective agents, especially those derived from natural sources, has gained increasing importance in clinical practice (Feng et al. 2022). Using UPLC–MS/MS analysis combined with the SIRIUS database and literature survey (Cui et al. 2024), 17 flavonoids were identified. Among them, vitexin was detected in both positive and negative ion modes in LC–MS analysis and was characterized as a high‐content flavonoid‐C‐glycoside with promising drug‐like properties. Through network pharmacology, potential pathways through which these flavonoids may alleviate HUA were predicted, including MAPK and PI3K‐Akt, with PPARγ/ABCG2 (PPARγ as a predicted upstream regulator, and ABCG2 as a downstream urate transporter) being one of the potential targeting mechanisms supported by recent findings (Han et al. 2025). It should be noted that, although the PPARγ‐ABCG2 regulatory pathway is already well established in urate regulation based on previous studies, the present study only hypothesizes that vitexin may act through this predicted axis, rather than providing causal proof. MAPK are closely linked to inflammation, oxidative stress, apoptosis, and kinase activity—all implicated in HUA pathogenesis (Kanellis et al. 2003; Li et al. 2016; Sautin and Johnson 2008). PI3K‐Akt signaling pathway may potentially enhance PPARγ/ABCG2‐mediated intestinal urate excretion, given that Akt acts as an upstream regulator of PPARγ by modulating its transcriptional activity and stability (Xiong et al. 2019; Kim et al. 2010).

Importantly, the novelty of this study does not primarily stem from proposing entirely new signaling pathways. Instead, it lies in the integration of phytochemical identification, computational prediction, and biological validation to establish a coherent mechanistic framework for vitexin. While pathways such as PI3K/Akt, PPARγ, and ABCG2 have been individually implicated in urate regulation and flavonoid pharmacology, they are often examined in isolation or inferred without direct linkage to chemically defined constituents.

PPARγ has been reported in previous studies as a key transcriptional regulator that may enhance uric acid excretion by activating the UA transporter ABCG2—a critical urate transporter highly expressed in both the kidneys and intestines—thereby potentially promoting uric acid excretion. These findings from prior studies suggest a regulatory relationship between PPARγ and ABCG2 in urate homeostasis. In the present study, this previously reported biological background was used as a rationale for further computational exploration rather than as an experimentally validated mechanism. Molecular docking indicated that vitexin could possibly bind to both PPARγ and ABCG2. Considering the predicted disease relevance, potential regulatory role, and mature target profile, PPARγ was selected for molecular dynamics simulations as a hypothesis‐supporting analysis to explore the stability and conformational characteristics of its interaction with vitexin at the atomic level. In contrast, ABCG2, as a downstream effector directly involved in urate transport, was prioritized for in vivo functional validation since changes in its expression provide a measurable physiological endpoint. This complementary strategy connects computational predictions with biological outcomes. As reflected by generally low RMSD and RMSF values, maintained secondary structure, persistent hydrogen‐bond interactions, and favorable binding free energy estimates, vitexin may form a relatively stable complex with PPARγ over the 100‐ns simulation.

Subsequent animal experiments demonstrated that vitexin exerts therapeutic effects against HUA. Mechanistically, it appears to act through multiple signaling pathways: suppressing inflammatory responses, downregulating key enzymes involved in purine metabolism to reduce uric acid production, and upregulating renal urate transporters—particularly ABCG2—to enhance uric acid excretion. Together, these multi‐target and multi‐pathway interactions form a coordinated therapeutic network. This systemic mode of action aligns with the observed in vivo efficacy of vitexin in maintaining metabolic homeostasis and alleviating tissue inflammation.

Importantly, the above integrated approach—beginning with LC‐MS‐based phytochemical analysis that identified vitexin as a primary interesting constituent of hawthorn total flavonoids, followed by network pharmacology prediction, and further validated through molecular docking and dynamics simulations—converged to indicate a possible involvement of the PPARγ–ABCG2 regulatory relationship as a plausible mechanistic hypothesis underlying the anti‐hyperuricemic effects of vitexin. The in silico findings were consistent with downstream biological validation of ABCG2, which demonstrated upregulated renal ABCG2 expression at both the transcriptional and translational levels and validated the pharmacological potential of vitexin (Xu et al. 2024). Moreover, this study offers systematic molecular‐level elucidation of vitexin's multi‐target and multi‐pathway regulatory mechanisms, providing valuable insights for the development of novel natural product‐based HUA therapeutics.

### 4.2 Multi‐Target Mechanisms of Vitexin in HUA

Vitexin effectively reduced systemic uric acid levels in mice by modulating the balance between uric acid production and excretion. It inhibited key enzymes in uric acid synthesis, including adenosine deaminase (ADA) and XOD, while upregulating renal excretory transporters ABCG2 and OAT1. Additionally, vitexin downregulated the reabsorptive transporter GLUT9 and enhanced ABCG2 expression in colonic tissues, thereby promoting uric acid elimination through both renal and intestinal pathways.

Given the close link between HUA and multi‐organ damage as well as systemic inflammation, vitexin's benefits extend beyond uric acid regulation. Accumulating evidence suggests that HUA contributes to hepatic and renal dysfunction, often accompanied by histopathological damage (Choe et al. 2015). In addition, approximately 30% of total uric acid is excreted via the intestinal tract, and HUA has been linked to impaired intestinal barrier integrity (Xu et al. 2016). Excessive uric acid accumulation in multiple tissues has also been associated with systemic inflammation and oxidative stress (Yip et al. 2020). Consistent with these pathological features, vitexin conferred substantial protection against HUA‐induced hepatic and renal injury, with particularly strong efficacy in restoring renal functional parameters. Specifically, it ameliorated histopathological injury in these target organs, preserved intestinal barrier integrity by upregulating tight junction proteins such as ZO‐1 and occludin, and significantly reduced plasma levels of inflammatory and oxidative markers, including IL‐6, IL‐1β, and MDA (An et al. 2023). These findings support a potential role for the gut in mediating the anti‐hyperuricemic effects of vitexin, particularly through upregulation of colonic ABCG2 and restoration of epithelial barrier integrity.

The above findings indicate that vitexin exerts comprehensive protective effects against HUA through multi‐target mechanisms involving uric acid metabolism, excretory transport, barrier maintenance, and inflammatory modulation (Chen et al. 2025). Notably, although numerous flavonoids have been reported to ameliorate hyperuricemia, many of these compounds exert their effects predominantly through single mechanisms, such as direct inhibition of xanthine oxidase or modulation of an individual urate transporter. In contrast, vitexin exhibited a coordinated multi‐level regulatory profile in this study. Specifically, vitexin not only suppressed key enzymes involved in uric acid production but also enhanced uric acid excretion through concurrent upregulation of ABCG2 in both renal and intestinal tissues. In addition, it restored intestinal barrier integrity and attenuated systemic inflammatory and oxidative responses, which are increasingly recognized as contributors to hyperuricemia‐associated organ injury. This integrated mode of action distinguishes vitexin from previously reported flavonoids and underscores its potential as a multi‐target natural candidate for hyperuricemia management.

### 4.3 Limitations and Future Perspectives

The present study has several limitations that should be acknowledged. First, the adenine and potassium oxonate–induced hyperuricemic mouse model may not fully replicate human hyperuricemia. In addition, plasma UA levels were not assessed during the early period (e.g., within 24 h) following PO discontinuation, and a transient spontaneous decline in UA levels during this interval cannot be excluded. Although plasma UA levels remained elevated throughout the treatment period (Figure 4C,D), these measurements primarily reflect sustained hyperuricemia during the intervention window rather than definitive long‐term model stability. More stable models, such as uricase‐knockout animals, could provide stronger translational relevance. In addition, while the PPARγ/ABCG2 pathway is well established in urate regulation based on previous studies, where PPARγ has been reported to transcriptionally regulate ABCG2 and thereby influence urate excretion, its role in this study was not experimentally validated. In this study, PPARγ was considered as a putative upstream regulatory factor derived from network pharmacology analysis and computational modeling, rather than a directly confirmed therapeutic target. In contrast, ABCG2 was selected for in vivo validation as a downstream urate transporter with direct functional relevance to uric acid excretion. Additionally, molecular dynamics simulation for ABCG2 was not performed. Histological evaluation was based on whole‐slide scans without dedicated high‐magnification fields. Moreover, the in vivo intervention employed only a single dose of vitexin without pharmacokinetic evaluation, limiting conclusions regarding dose–response and systemic exposure. Oral bioavailability of vitexin in mice is reported to be low (approximately 3.9%–4.9%) (Wang et al. 2013), and flavonoids generally exhibit variable absorption (Mustapha and Taib 2023). Furthermore, although intestinal ABCG2 expression and barrier integrity were assessed, other gut‐related mechanisms, including microbiota composition and functional urate transport, remain to be explored. Despite these limitations, the integrated analysis, computational prediction, and in vivo findings provide a mechanistic framework supporting the potential of vitexin as a multi‐target therapeutic candidate for hyperuricemia.

## 5 Conclusion

In this study, vitexin was identified from the TCM herb hawthorn—known for its spleen‐strengthening properties—through UPLC‐MS/MS and in silico approaches, including network pharmacology, molecular docking, and molecular dynamics simulations, which predicted potential targets and mechanisms. These approaches suggested that vitexin may reduce uric acid by targeting PPARγ and subsequently modulating the urate transporter ABCG2, a hypothesis that was explored in vivo. Systematic animal experiments, using the clinically approved uricosuric agent benzbromarone as a reference, validated the multi‐target efficacy and proposed mechanisms of vitexin. These experiments further demonstrated that vitexin effectively alleviates HUA‐related pathology by maintaining uric acid homeostasis, reducing systemic inflammation, and protecting against organ damage. The observed therapeutic effects of vitexin likely involve a synergistic combination of actions, including inhibiting uric acid synthesis, promoting uric acid excretion, and mitigating oxidative stress and inflammation, with evidence supporting involvement of intestinal ABCG2 and barrier integrity. Overall, these findings highlight vitexin as a promising natural candidate for HUA management, with potential applications in pharmaceutical development and functional nutrition.

## Author Contributions

**Kai‐Wen Kang:** investigation, validation, software, methodology, formal analysis. **Shu‐Yu Wu:** writing – original draft, visualization, methodology, investigation, validation. **Hao Zheng:** investigation, data curation. **Hui‐Qing Lv:** data curation, validation, resources. **Cheng‐Ping Wen:** validation, funding acquisition, formal analysis. **Shu‐Ying Li:** validation, methodology. **Ri‐Hui Wu:** data curation, supervision. **Wan‐Bin Song:** validation, formal analysis, conceptualization. **Xiao Yuan:** writing – review and editing, funding acquisition, validation, formal analysis. **Jing Chen:** visualization, validation, formal analysis. **Li‐She Gan:** writing – review and editing, funding acquisition, validation, formal analysis, conceptualization. **Dan‐Yu Huang:** data curation, validation, formal analysis.

## Funding

This work was supported by the National Natural Science Foundation of China (22077111), the Guangdong and Macao cooperation project from Department of Science and Technology of Guangdong Province and Jiangmen Science and Technology Bureau (2022A0505020026, 2024A0505090024), the Department of Education of Guangdong Province (2024ZDZX4015), and the Key Research and Development Program of Zhejiang Province (2023C03040).

## Ethics Statement

Animal experiments were approved by the Animal Experiment Ethics Committee of the International Healthcare Innovation Institute (Jiangmen) (registration number: N2023021) and conducted in accordance with the Animal Center's Guidelines for the Care and Use of Laboratory Animals.

## Conflicts of Interest

The authors declare no conflicts of interest.

## Supporting information


**Table S1:** Primer sequences used in this study.
**Table S2:** Chromatographic elution program.
**Table S3:** Supplementary materials for the identification of major flavonoids in tfh by LC–MS^n^.
**Table S4:** Bioavailability and drug‐likeness assessment of selected flavonoids based on SwissADME analysis.
**Figure S1:** Secondary mass spectra for the identification of major flavonoids in TFH using LC–MS^n^.
**Figure S2:** Three‐dimensional binding pose of MRL24 within the PPARγ binding site, with an enlarged view of the interaction environment.
**Figure S3:** Original Western blot images.

## Data Availability

The data that support the findings of this study are available from the corresponding author upon reasonable request.
