# Supplementary material for: Antihyperuricemic Effect of Hawthorn Flavonoid Vitexin: LC–MS Analysis, Network Pharmacology, and In Vivo Verification of Molecular Mechanisms
Source: Food Sci Nutr. 2026 May 27;14(6):e71925. doi: 10.1002/fsn3.71925 (PMC13239997; doi:10.1002/fsn3.71925)

**Table S1.** Primer sequences used in this study

| Gene name | Forward primer (5'-3') | Reverse primer (5'-3') |
| --- | --- | --- |
| GAPDH | AGTGTTTCCTCGTCCCGTAG | GCCGTGAGTGGAGTCATACT |
| ABCG2 | ACTCCCCATGAGGTTCTTGCC | ACTGGCCGTATAAGCCACCAT |
| OAT1 | TGCTCCCCTACTGCTGATG | GAGGCAAGATTCGGGTCGTC |
| Occludin | AGCCTCGGTACAGCAGCAAT | CCCACCTGTCGTGTAGTCTGT |
| ZO-1 | CCACCTCTGTCCAGCTCTTC | CACCGGAGTGATGGTTCT |

**Table S2.** Chromatographic elution program

| Time(min) | Flow(mL/min) | %A | %B |
| --- | --- | --- | --- |
| initial | 0.300 | 98.0 | 2.0 |
| 2.00 | 0.300 | 95.0 | 5.0 |
| 10.00 | 0.300 | 85.0 | 15.0 |
| 15.00 | 0.300 | 75.0 | 25.0 |
| 18.00 | 0.300 | 50.0 | 50.0 |
| 23.00 | 0.300 | 0.0 | 100.0 |
| 25.00 | 0.300 | 98.0 | 2.0 |
| 30.00 | 0.300 | 98.0 | 2.0 |

**Table S3.** Supplementary Materials for the Identification of Major Flavonoids in TFH by LC–MS^n^

| Compounds | RT (min) | Charge state | Formula | Name | Predicted (m/z) | Main fragments (m/z) | Chemical Structures | SMILES |
| --- | --- | --- | --- | --- | --- | --- | --- | --- |
| 1 | 13.34 | [M+H] | C_33_H_40_O_20_ | Quercetin-3-*O*-[2,6-di-*α*-L-rhamnopyranosyl-*β*-D-galactopyranoside] | 757.2113 | 611.1614, 465.1019,  303.0502 |  | CC1[C@@H]([C@@H](C([C@@H](O1)O[C@H]2[C@H](C(O[C@H](C2O)OCC3[C@@H](C(C([C@@H](O3)OC4=C(OC5=CC(=CC(=C5C4=O)O)O)C6=CC(=C(C=C6)O)O)O)O)O)C)O)O)O)O |
| 2 | 14.12 | [M+H] | C_27_H_30_O_16_ | Rutin | 611.1534 | 465.1026, 303.0493 |  | C[C@H]1[C@@H]([C@H]([C@H]([C@@H](O1)OC[C@@H]2[C@H]([C@@H]([C@H]([C@@H](O2)OC3=C(OC4=CC(=CC(=C4C3=O)O)O)C5=CC(=C(C=C5)O)O)O)O)O)O)O)O |
| 3 | 14.82 | [M+H] | C_21_H_20_O_10_ | Vitexin | 433.1056 | 415.3347, 397.0910, 283.2582 |  | C1=CC(=CC=C1C2=CC(=O)C3=C(O2)C(=C(C=C3O)O)[C@H]4[C@@H]([C@H]([C@@H]([C@H](O4)CO)O)O)O)O |
| 4 | 14.82 | [M+Na] | C_21_H_20_O_10_ | Isovitexin | 455.1056 | 365.0648, 303.0636 |  | C1=CC(=CC=C1C2=CC(=O)C3=C(O2)C=C(C(=C3O)[C@H]4[C@@H]([C@H]([C@@H]([C@H](O4)CO)O)O)O)O)O |
| 5 | 15.03 | [M+H] | C_21_H_20_O_12_ | Hyperoside | 465.0955 | 303.0513, 289.0697 |  | C1=CC(=C(C=C1C2=C(C(=O)C3=C(C=C(C=C3O2)O)O)O[C@H]4[C@@H]([C@H]([C@H]([C@H](O4)CO)O)O)O)O)O |
| 6 | 15.67 | [M+H] | C_20_H_20_O_8_ | 5-Hydroxyauranetin | 389.1158 | 281.0490, 192.0369 |  | COC1=CC=C(C=C1)C2=C(C(=O)C3=C(C(=C(C(=C3O2)OC)OC)OC)O)OC |
| 7 | 10.56 | [M+H] | C_45_H_38_O_18_ | Procyanidin C1 | 867.2058 | 715.1837,  579.1505 |  | C1[C@H]([C@H](OC2=C1C(=CC(=C2[C@@H]3[C@H]([C@H](OC4=C(C(=CC(=C34)O)O)[C@@H]5[C@H]([C@H](OC6=CC(=CC(=C56)O)O)C7=CC(=C(C=C7)O)O)O)C8=CC(=C(C=C8)O)O)O)O)O)C9=CC(=C(C=C9)O)O)O |
| 8 | 14.12 | [M+H] | C_39_H_32_O_15_ | Kandelin A1 | 741.1741 | 589.1351, 451.1027,  289.0705 |  | C1[C@@H]([C@H](OC2=C1C(=CC(=C2[C@@H]3[C@H]([C@H](OC4=C3C(=CC5=C4[C@H](CC(=O)O5)C6=CC(=C(C=C6)O)O)O)C7=CC(=C(C=C7)O)O)O)O)O)C8=CC(=C(C=C8)O)O)O |
| 9 | 12.18 | [M−H] | C_27_H_30_O_15_ | Kaempferol 3-neohesperidoside | 593.1494 | 473.1110, 353.0659 |  | C[C@H]1[C@@H]([C@H]([C@H]([C@@H](O1)O[C@@H]2[C@H]([C@@H]([C@H](O[C@H]2OC3=C(OC4=CC(=CC(=C4C3=O)O)O)C5=CC=C(C=C5)O)CO)O)O)O)O)O |
| 10 | 14.46 | [M−H] | C_27_H_30_O_15_ | Vitexin-4″-*O*-*β*-D-glucopyranoside | 593.1494 | 413.0871, 293.0451 |  | C1=CC(=CC=C1C2=CC(=O)C3=C(O2)C(=C(C=C3O)O)[C@H]4[C@@H]([C@H]([C@@H]([C@H](O4)CO)O[C@H]5[C@@H]([C@H]([C@@H]([C@H](O5)CO)O)O)O)O)O)O |
| 11 | 14.78 | [M−H] | C27H30O14 | Vitexin-2″-*O*-*α*-L-rhamnopyranoside | 577.1539 | 413.0871, 293.0451 |  | C[C@H]1[C@@H]([C@H]([C@H]([C@@H](O1)O[C@@H]2[C@H]([C@@H]([C@H](O[C@H]2C3=C(C=C(C4=C3OC(=CC4=O)C5=CC=C(C=C5)O)O)O)CO)O)O)O)O)O |
| 12 | 15.04 | [M−H] | C_21_H_20_O_12_ | Spiraeoside | 463.0955 | 300.0289, 178.9986 |  | C1=CC(=C(C=C1C2=C(C(=O)C3=C(C=C(C=C3O2)O)O)O)O)O[C@H]4[C@@H]([C@H]([C@@H]([C@H](O4)CO)O)O)O |
| 13 | 18.80 | [M−H] | C_15_H_10_O_6_ | Luteolin | 285.0477 | 151.0041, 133.0300 |  | C1=CC(=C(C=C1C2=CC(=O)C3=C(C=C(C=C3O2)O)O)O)O |
| 14 | 18.81 | [M−H] | C_15_H_10_O_7_ | Quercetin | 301.0427 | 178.9977, 151.0041 |  | C1=CC(=C(C=C1C2=C(C(=O)C3=C(C=C(C=C3O2)O)O)O)O)O |
| 15 | 11.49 | [M−H] | C_15_H_12_O_7_ | (+)-Taxifolin | 303.0583 | 285.0405, 125.0232 |  | C1=CC(=C(C=C1[C@@H]2[C@H](C(=O)C3=C(C=C(C=C3O2)O)O)O)O)O |
| 16 | 11.49 | [M+Cl] | C_15_H_14_O_6_ | Catechin | 325.079 | 245.0806, 125.0235 |  | C1[C@@H]([C@H](OC2=CC(=CC(=C21)O)O)C3=CC(=C(C=C3)O)O)O |
| 17 | 14.09 | [M−H] | C_30_H_26_O_12_ | Procyanidin B2 | 577.1424 | 289.0711, 245.0457, 161.0234 |  | C1[C@H]([C@H](OC2=C1C(=CC(=C2[C@@H]3[C@H]([C@H](OC4=CC(=CC(=C34)O)O)C5=CC(=C(C=C5)O)O)O)O)O)C6=CC(=C(C=C6)O)O)O |

**Table S4. Bioavailability and drug-likeness assessment of selected flavonoids based on SwissADME analysis**

| Druglikeness | Spiraeoside | Hyperoside | Vitexin |
| --- | --- | --- | --- |
| Lipinski | No; 2 violations: NorO>10, NHorOH>5 | No; 2 violations: NorO>10, NHorOH>5 | Yes; 1 violation: NHorOH>5 |
| Ghose | No; 1 violation: WLOGP<-0.4 | No; 1 violation: WLOGP<-0.4 | Yes |
| Veber | No; 1 violation: TPSA>140 | No; 1 violation: TPSA>140 | No; 1 violation: TPSA>140 |
| Egan | No; 1 violation: TPSA>131.6 | No; 1 violation: TPSA>131.6 | No; 1 violation: TPSA>131.6 |
| Muegge | No; 3 violations: TPSA>150, H-acc>10, H-don>5 | No; 3 violations: TPSA>150, H-acc>10, H-don>5 | No; 2 violations: TPSA>150, H-don>5 |
| Bioavailability Score | 0.17 | 0.17 | 0.55 |


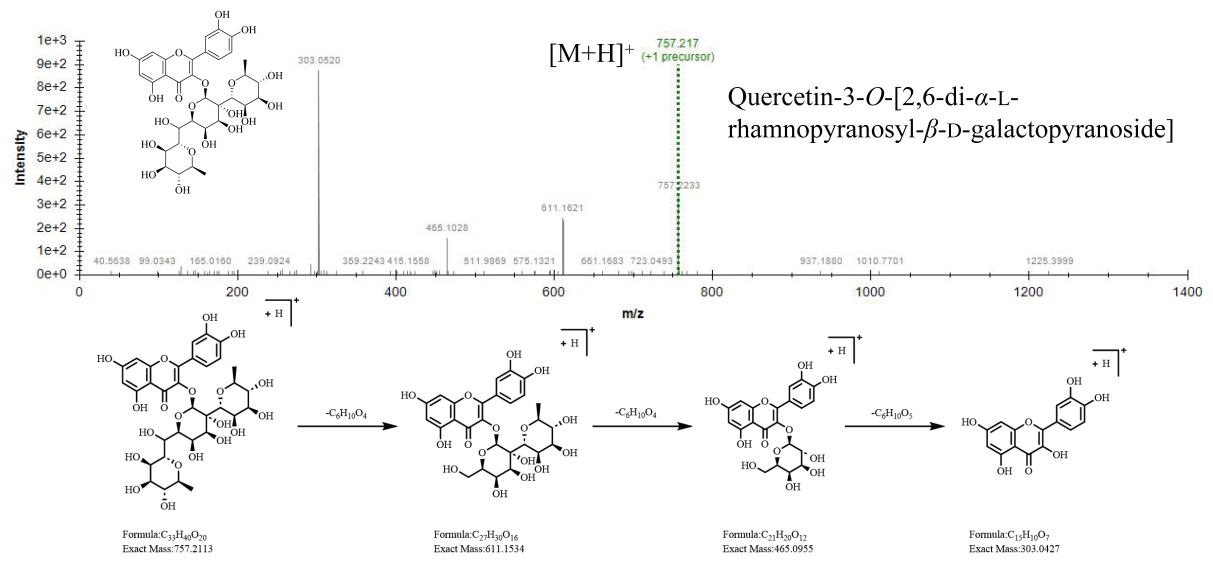

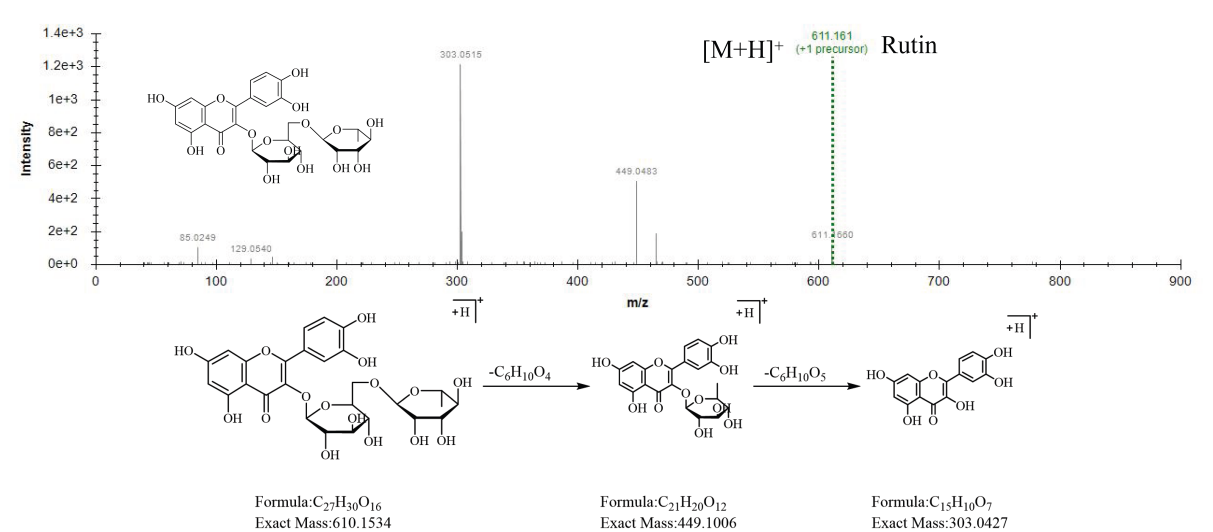

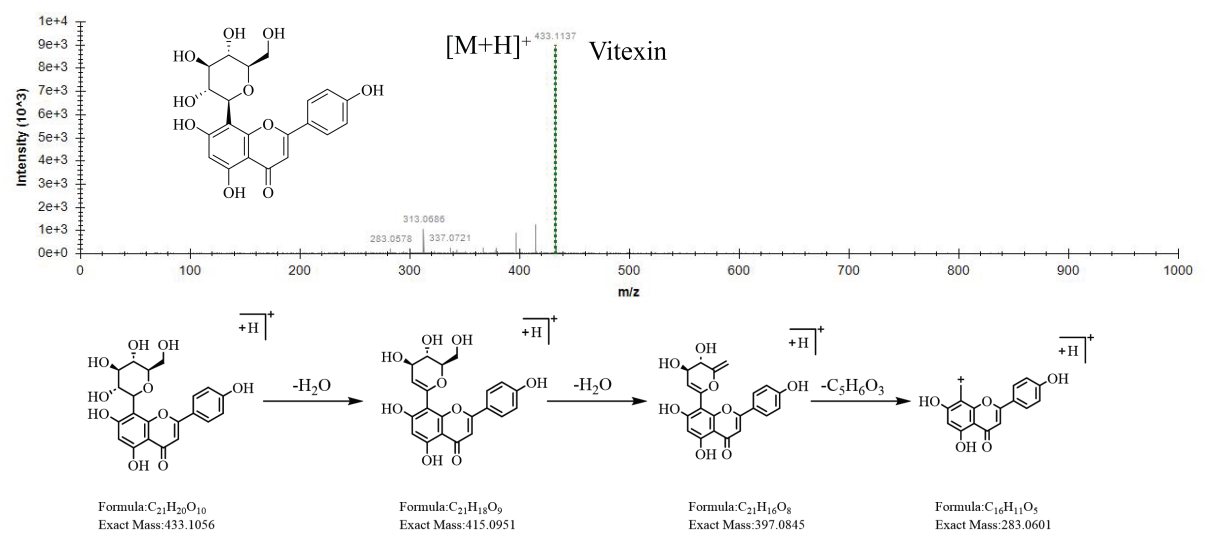

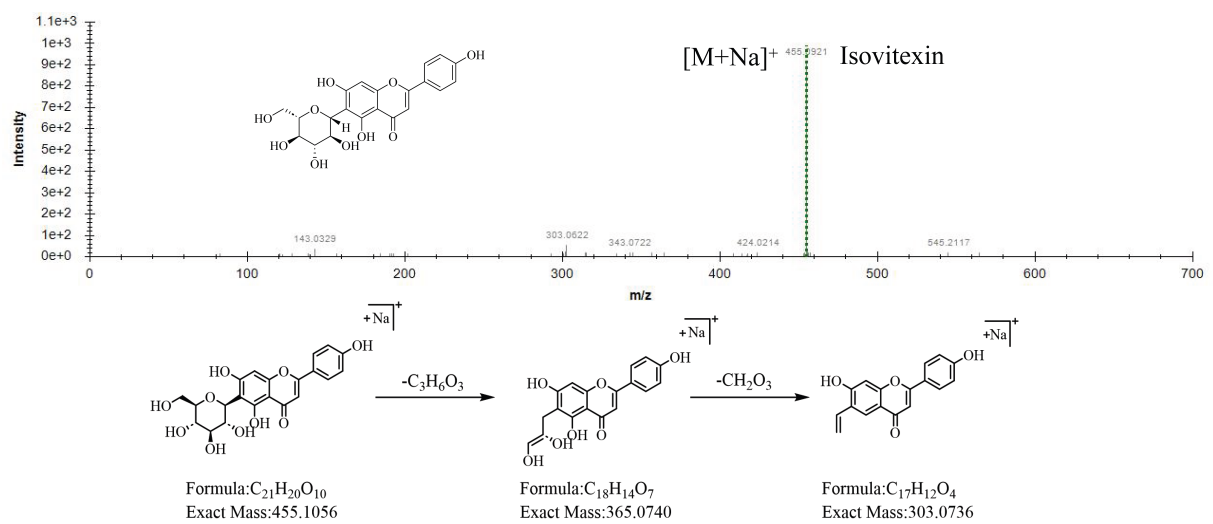

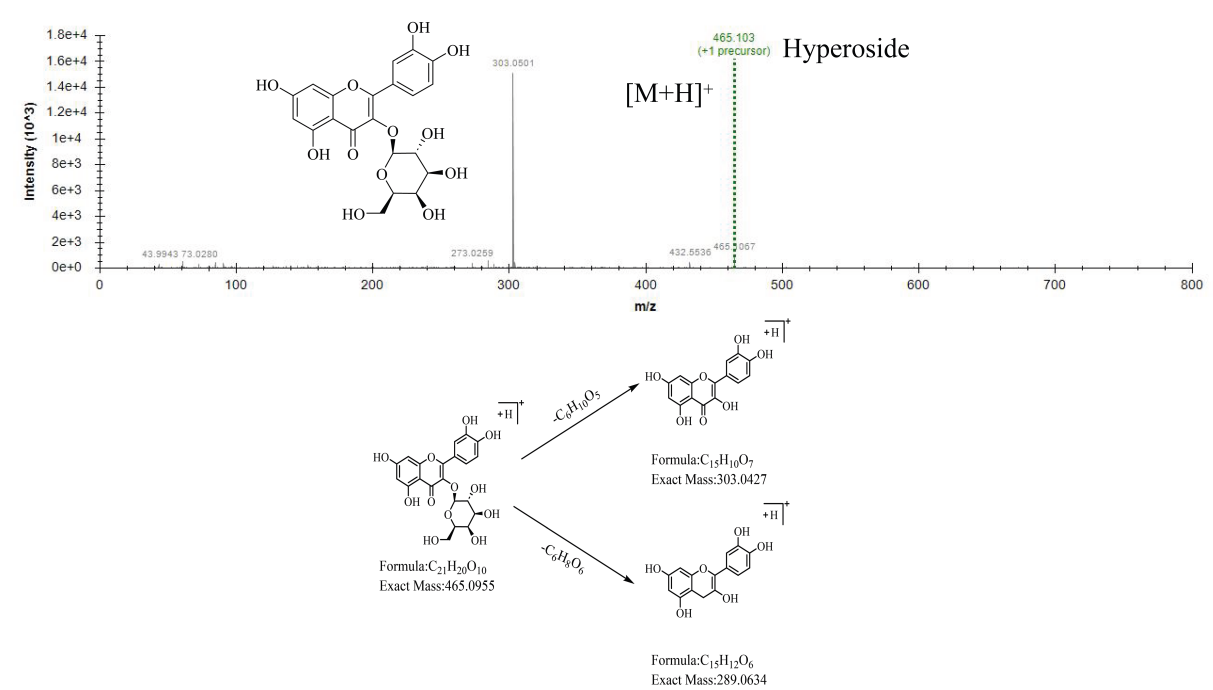

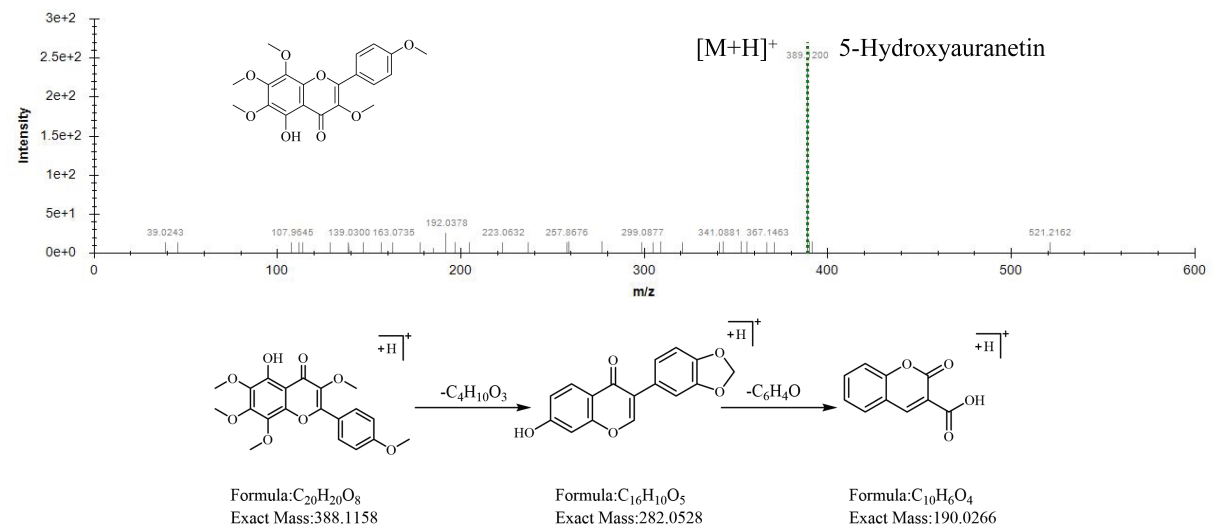

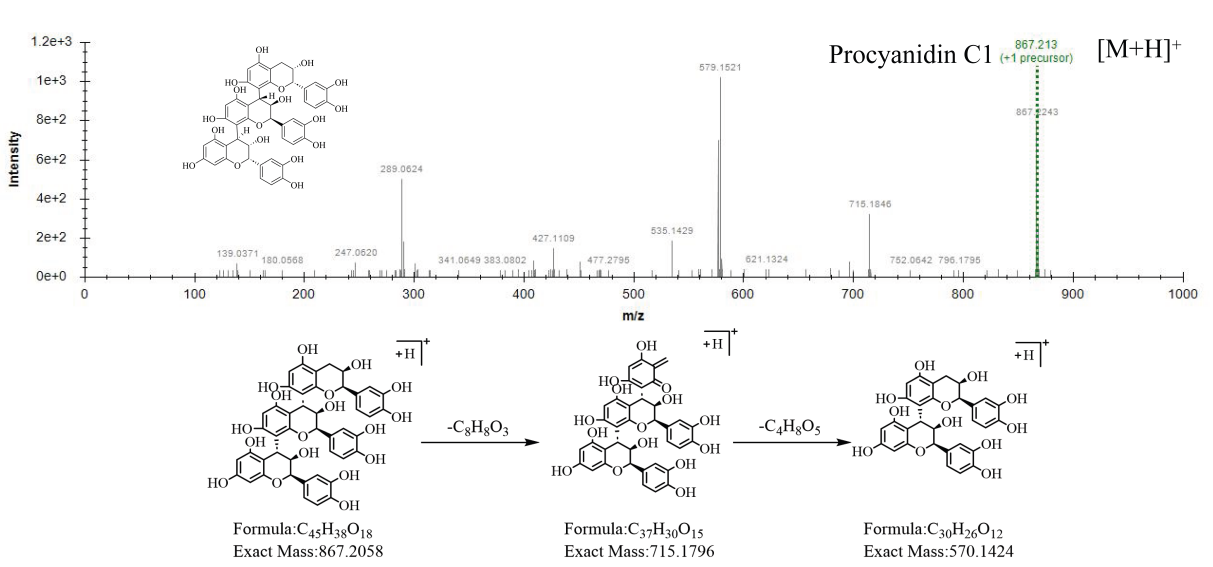

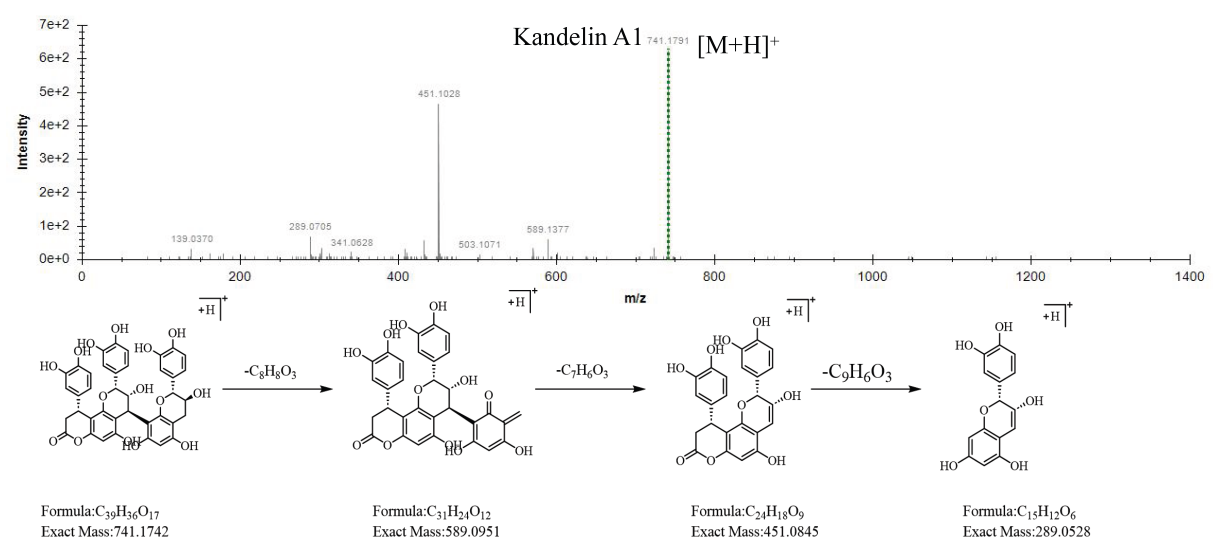

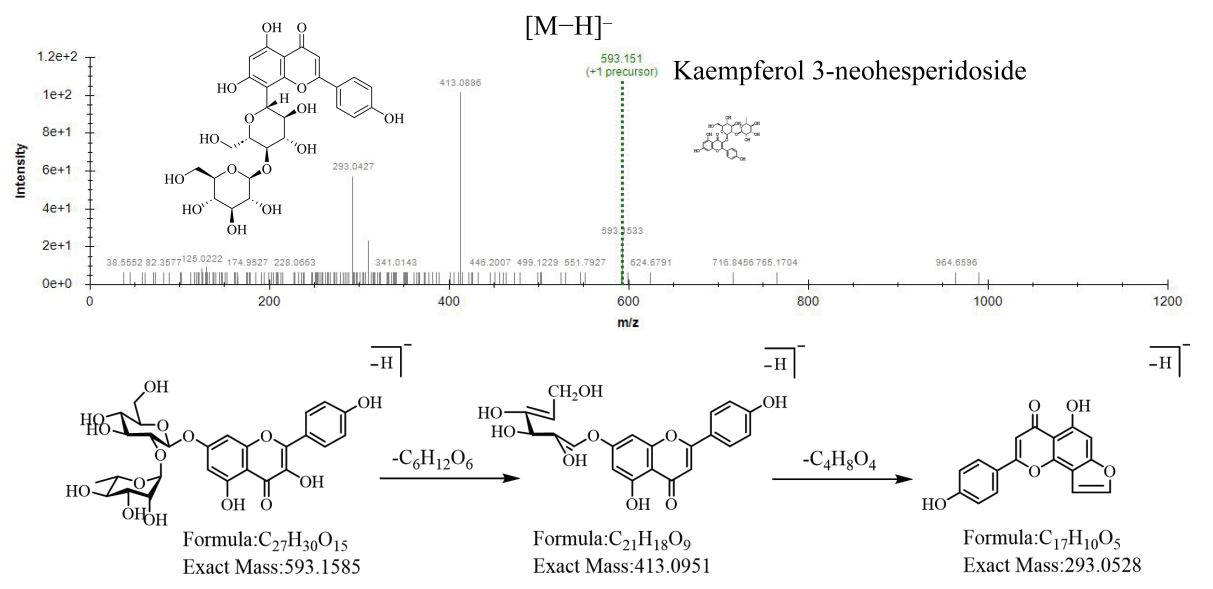

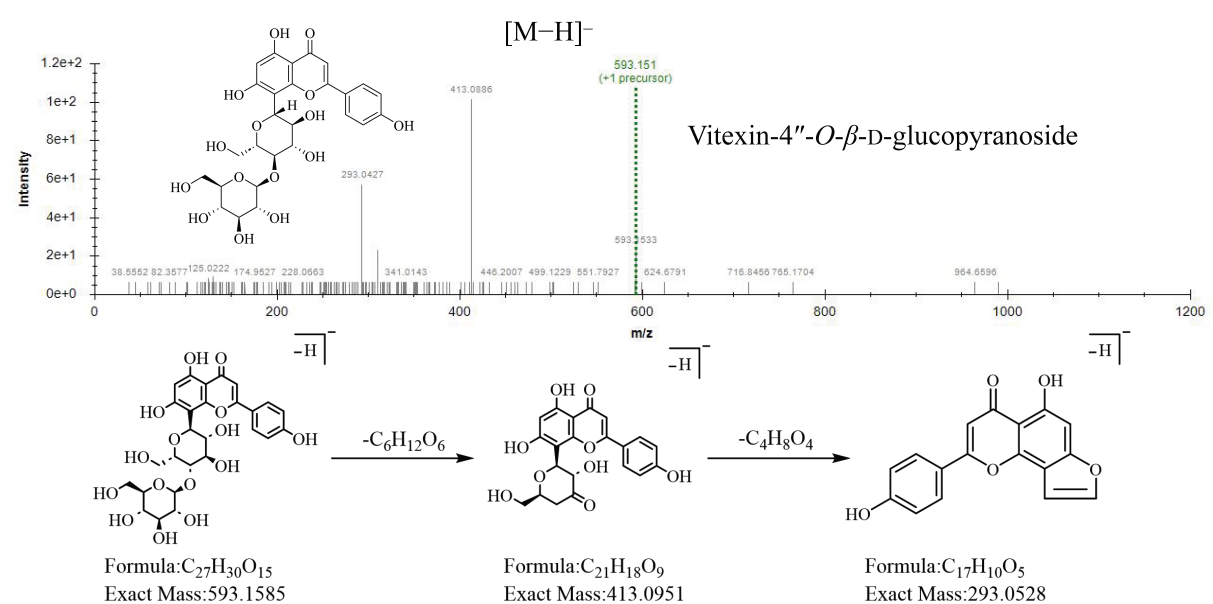

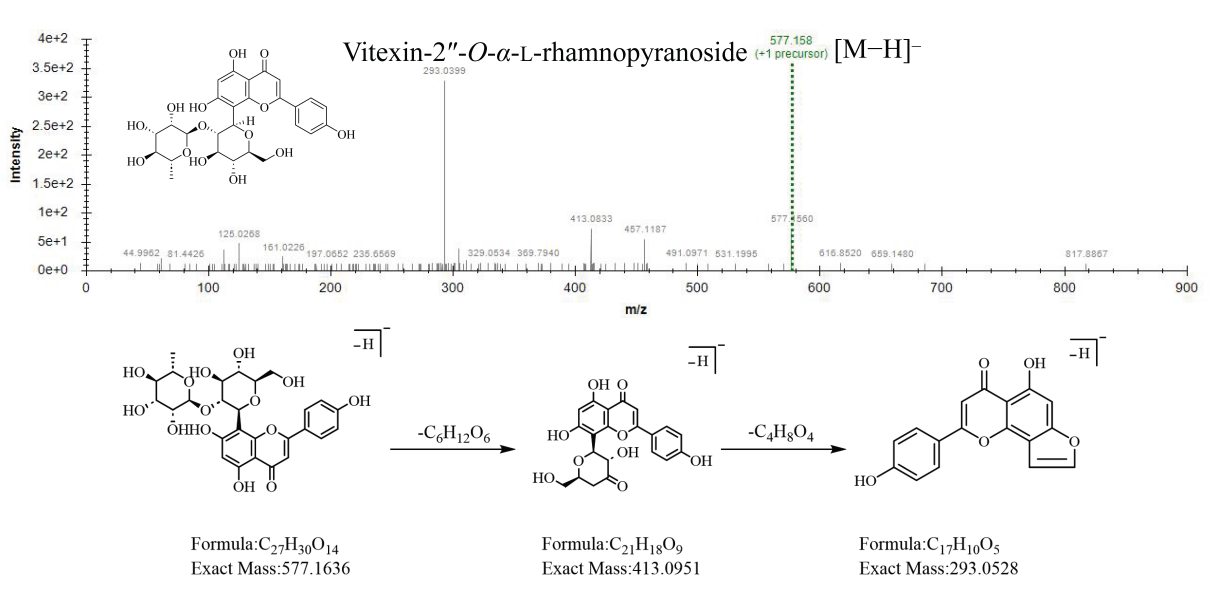

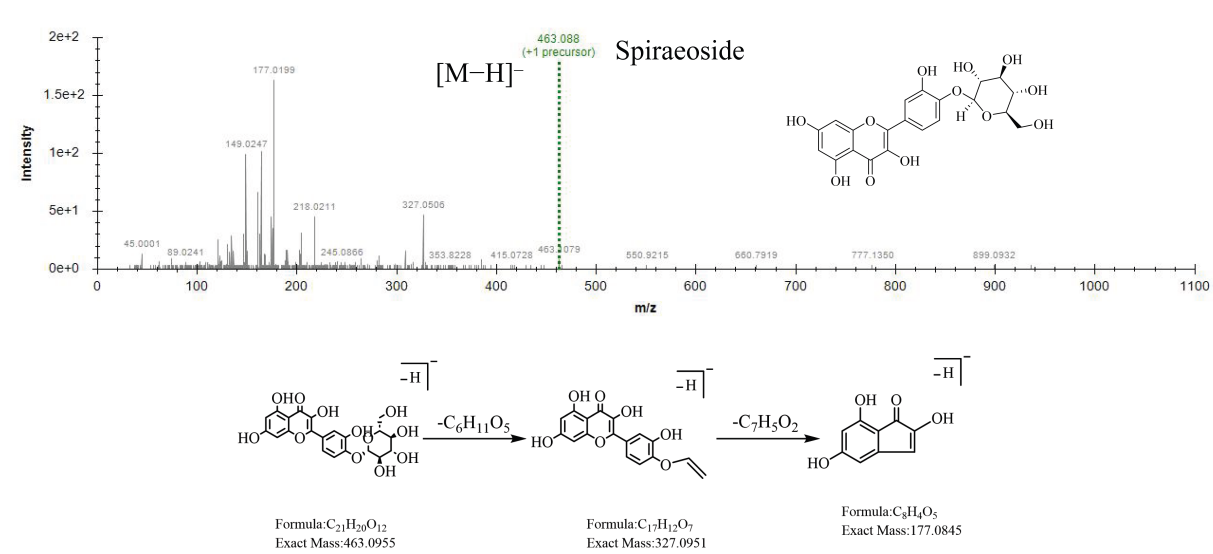

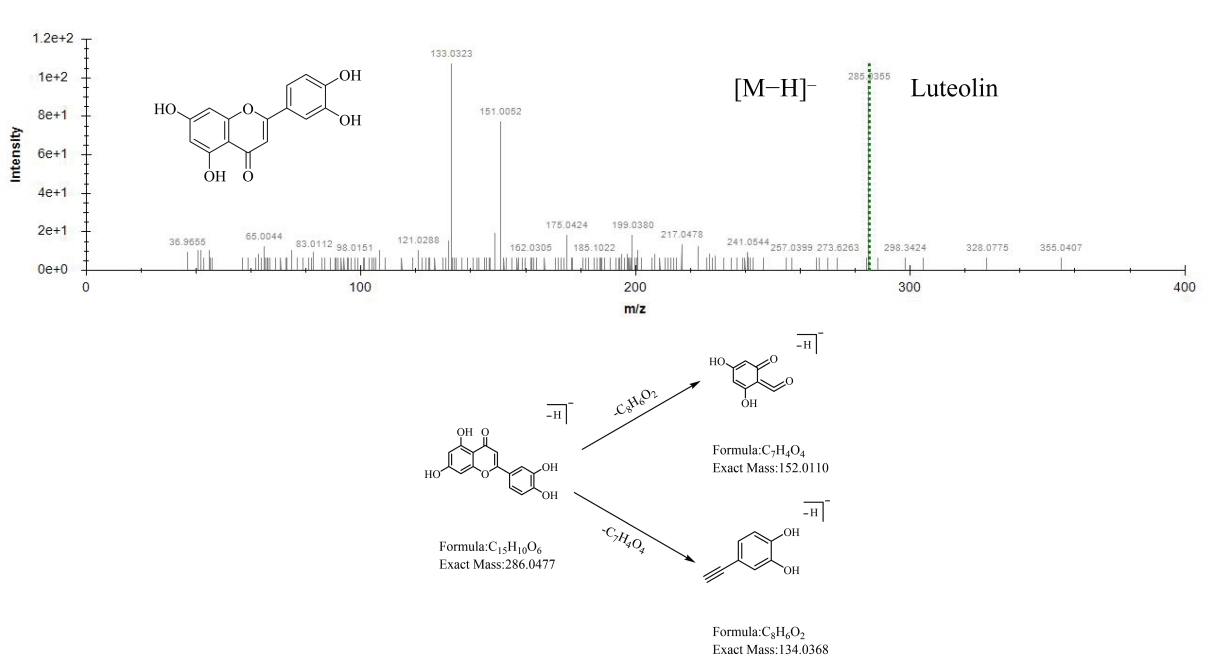

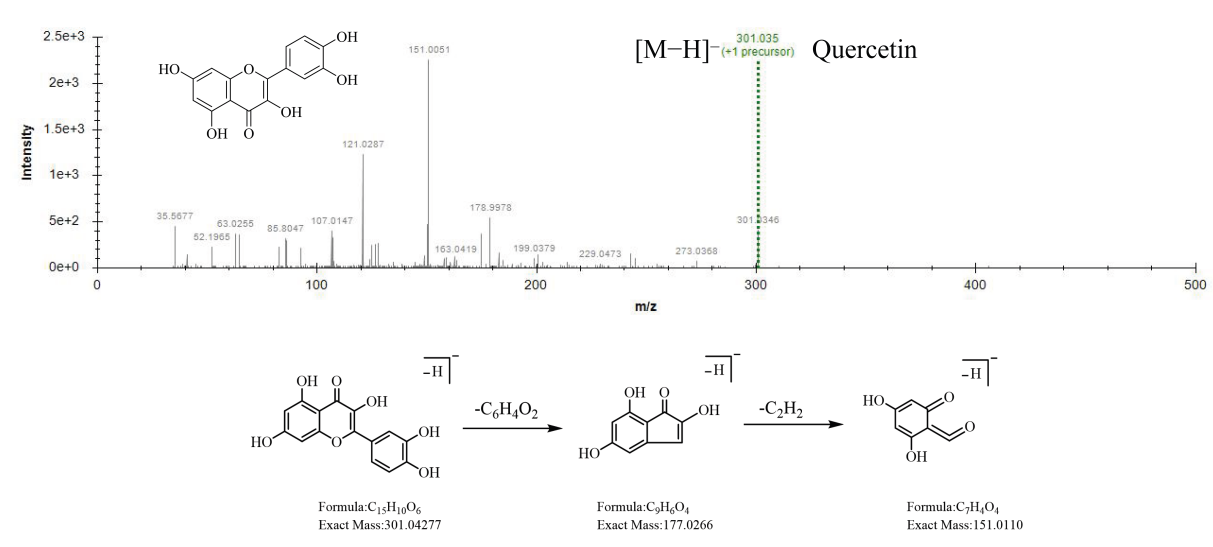

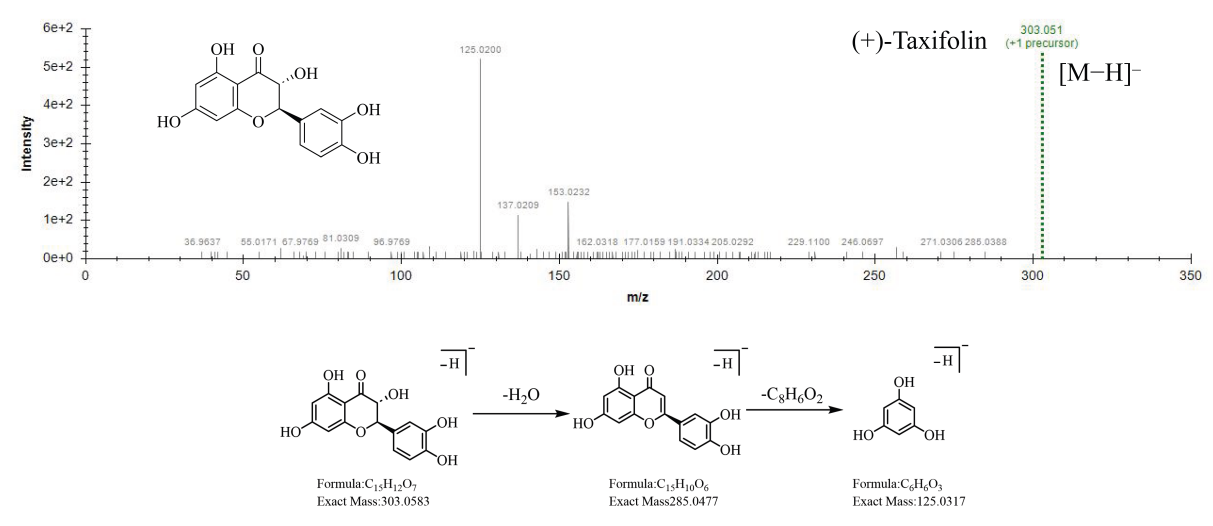

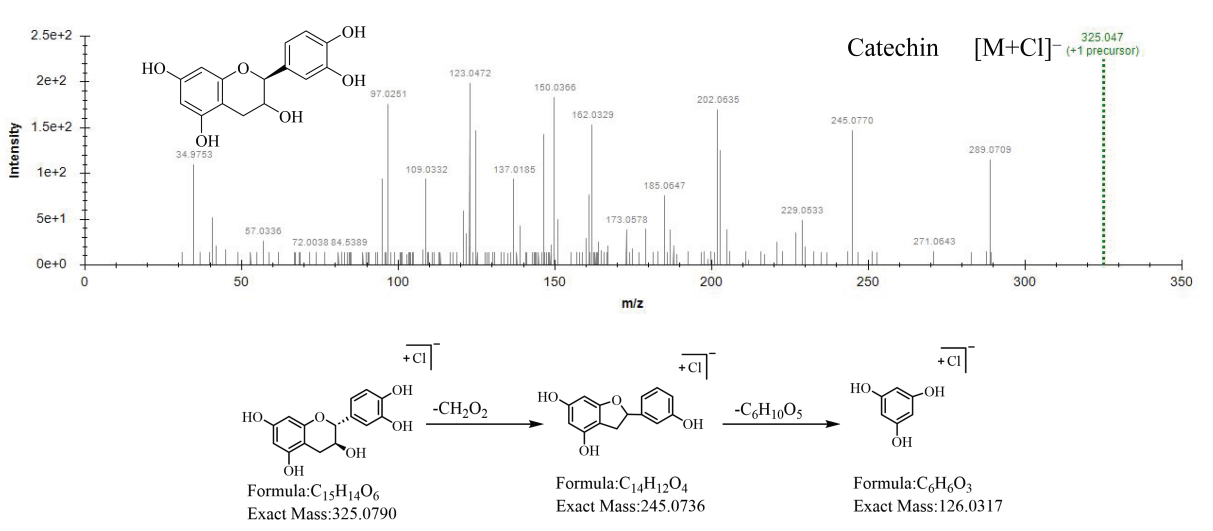

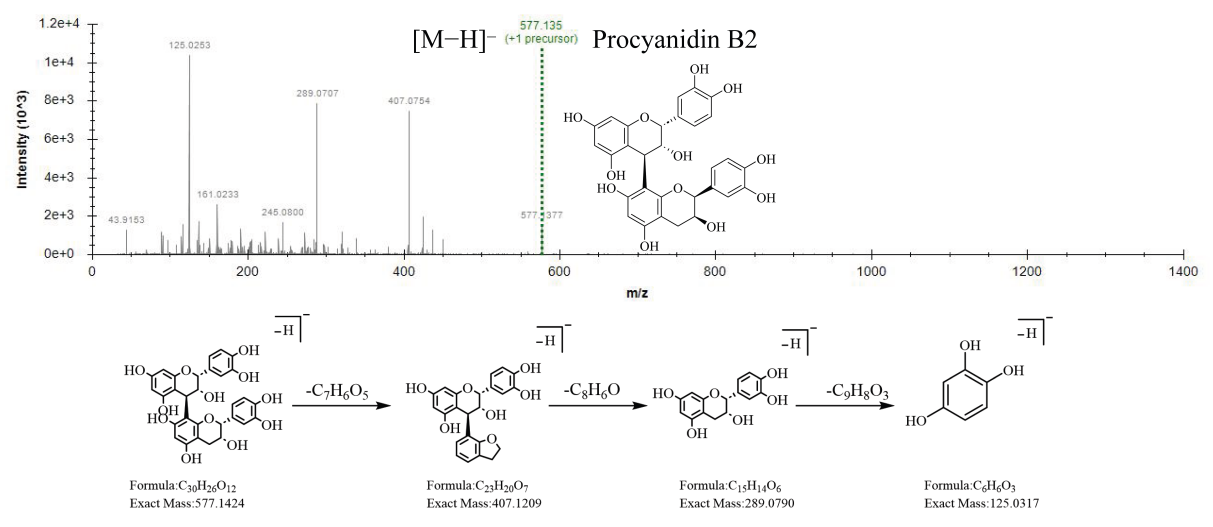


**Figure S1.** Secondary mass spectra for the identification of major flavonoids in TFH using LC–MS^n^


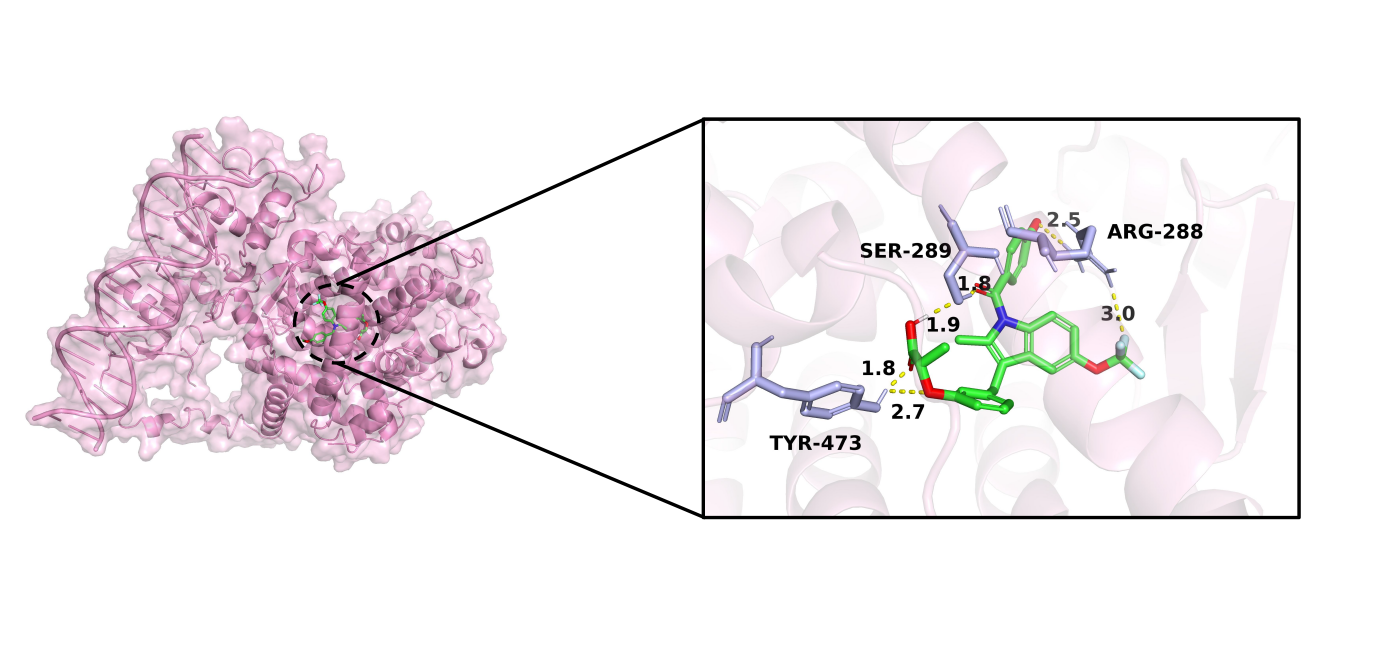


**Figure S2.** Three-dimensional binding pose of MRL24 within the PPARγ binding site, with an enlarged view of the interaction environment.

**Figure S3.** Original Western blot Images

**Figure 5E**

ABCG2 in mouse kidney:


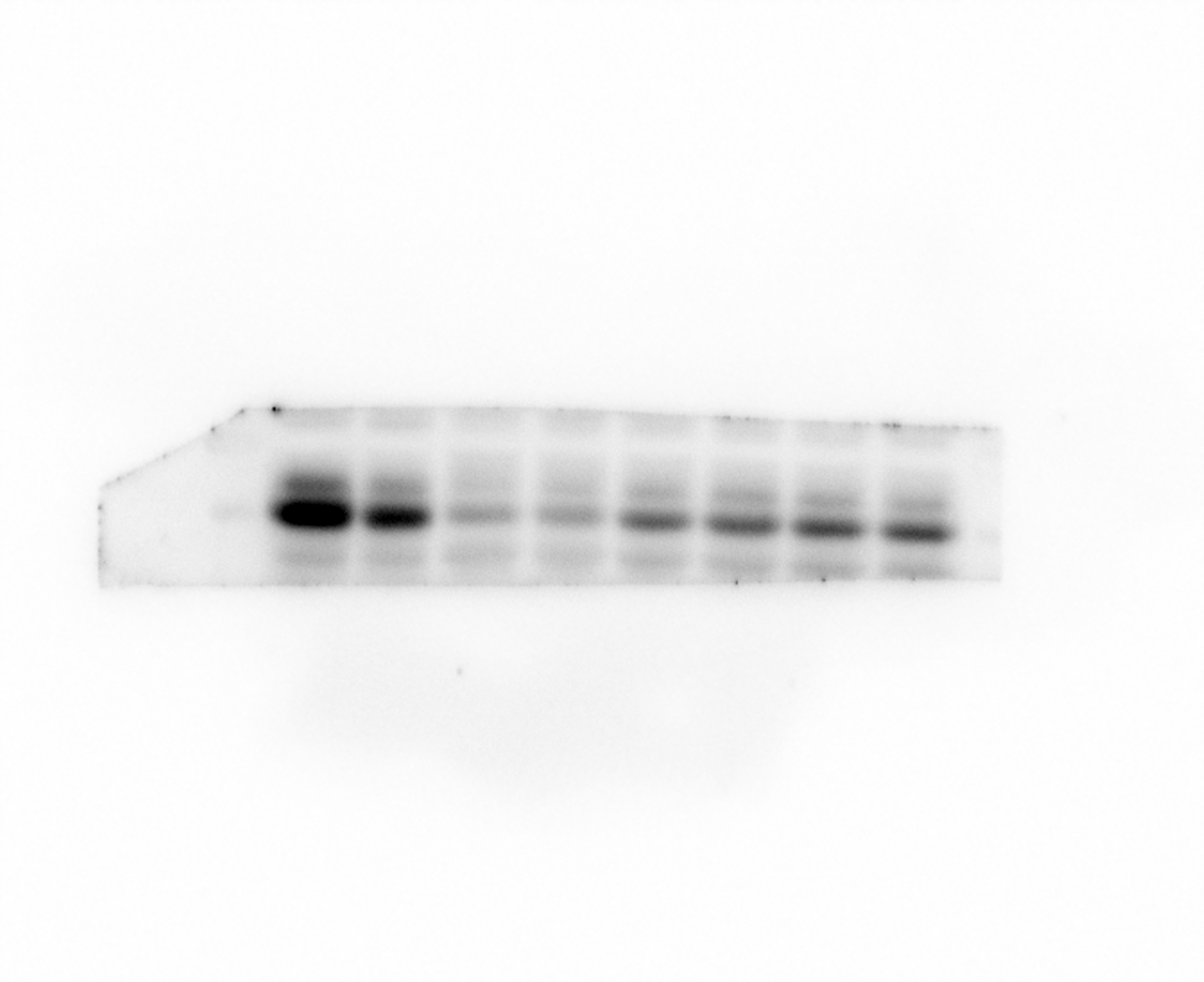


**55**

**kDa**

**56**

GAPDH:


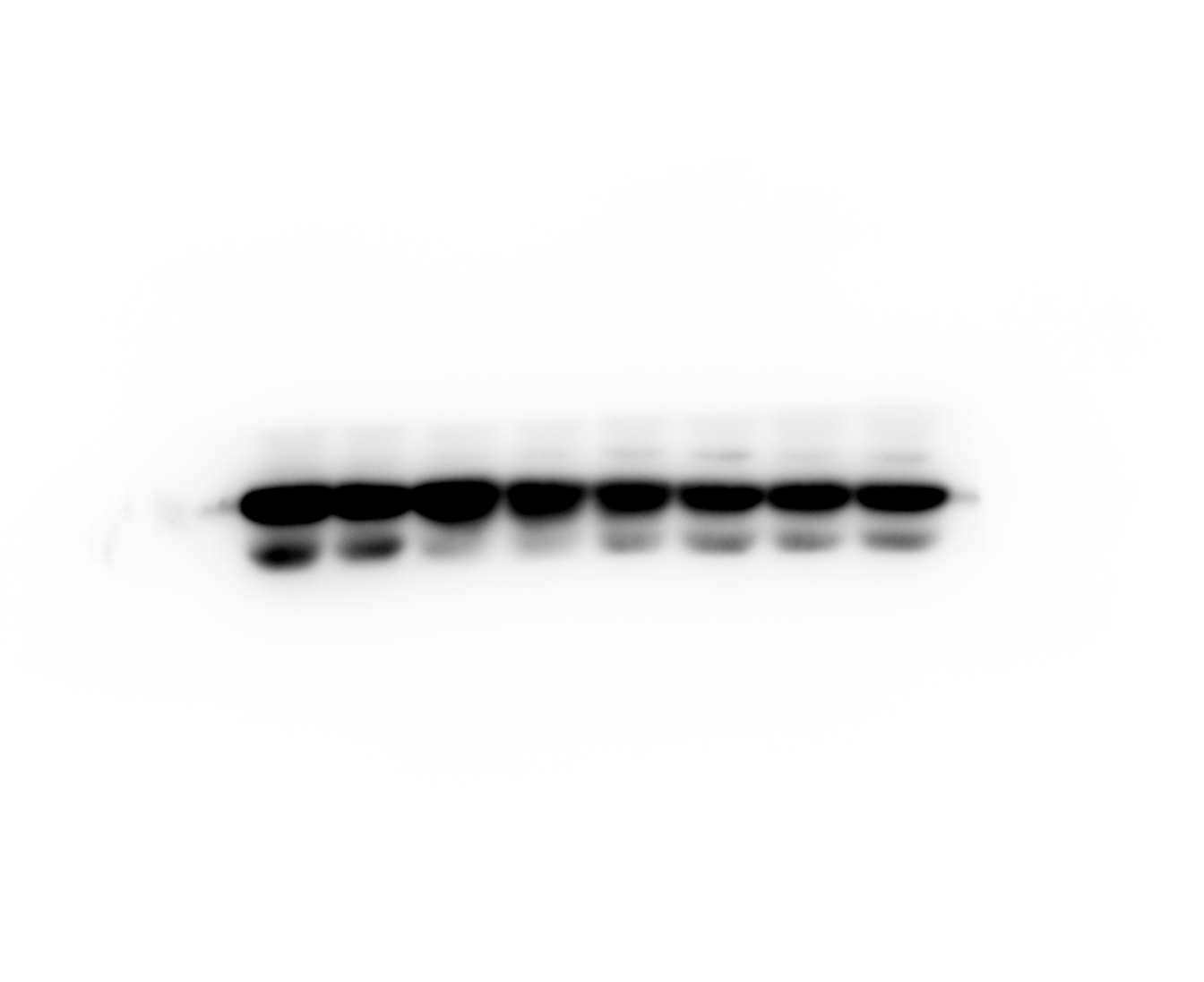


ABCG2 in mouse kidney:


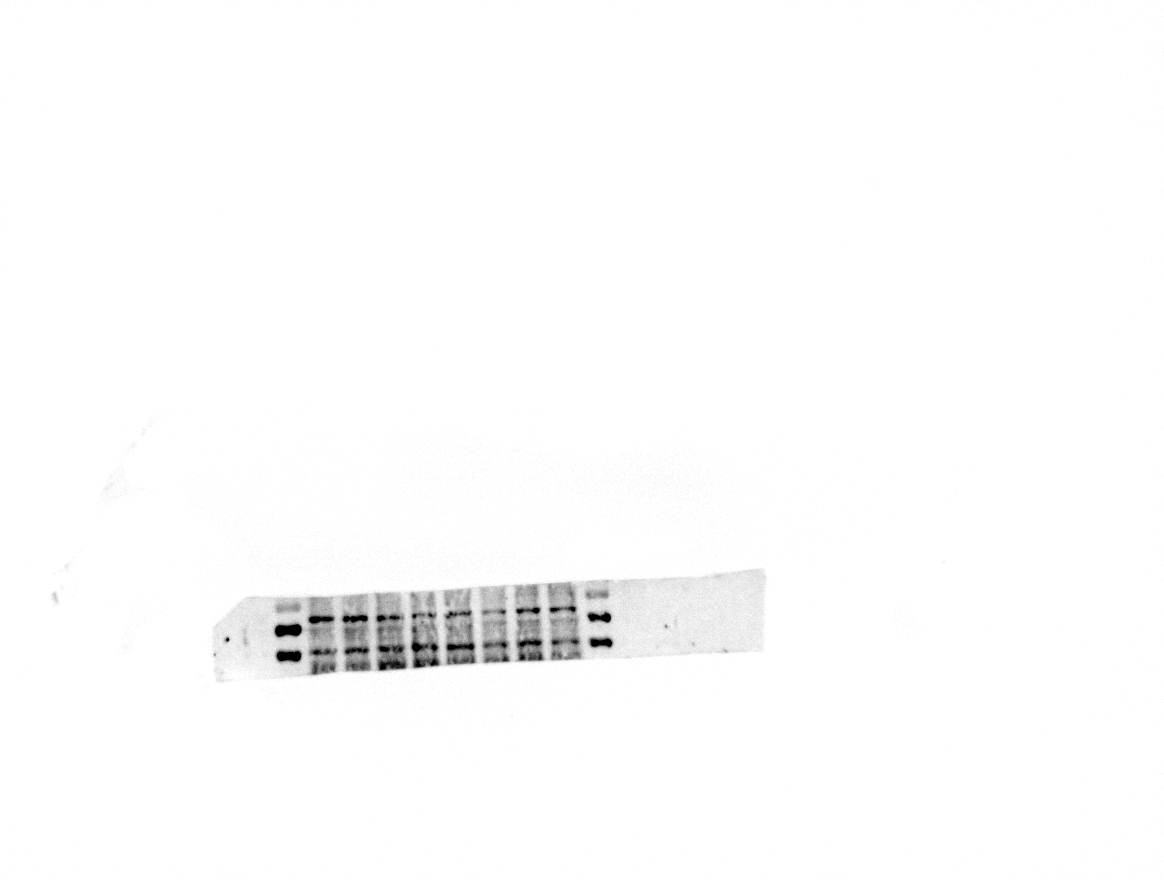


**56**

**70**

**55**

**kDa**

**100**

GAPDH


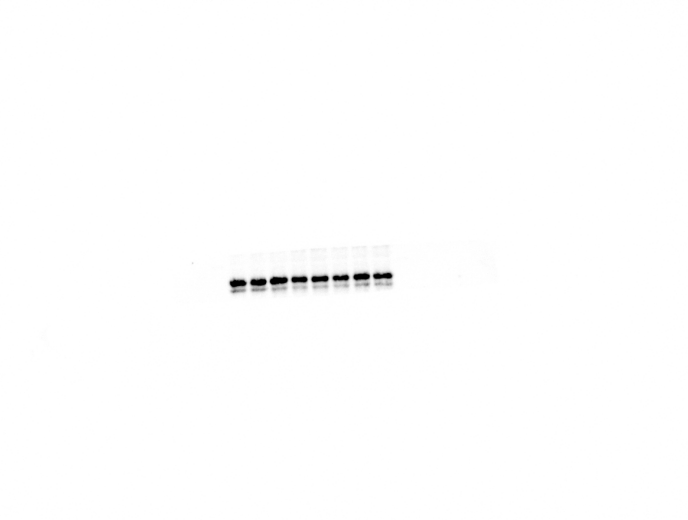


OAT1 in mouse kidney


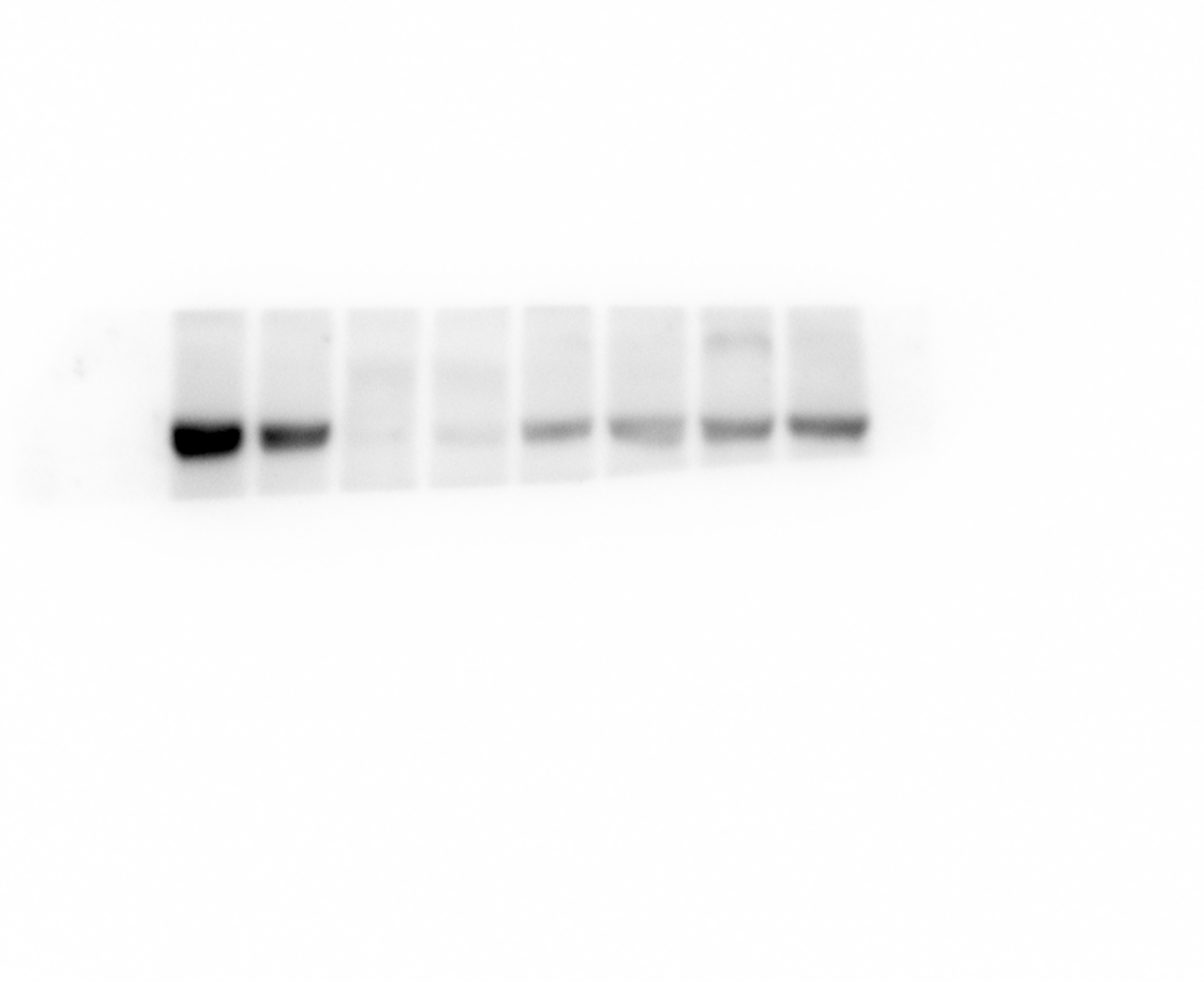


**kDa**

**55**


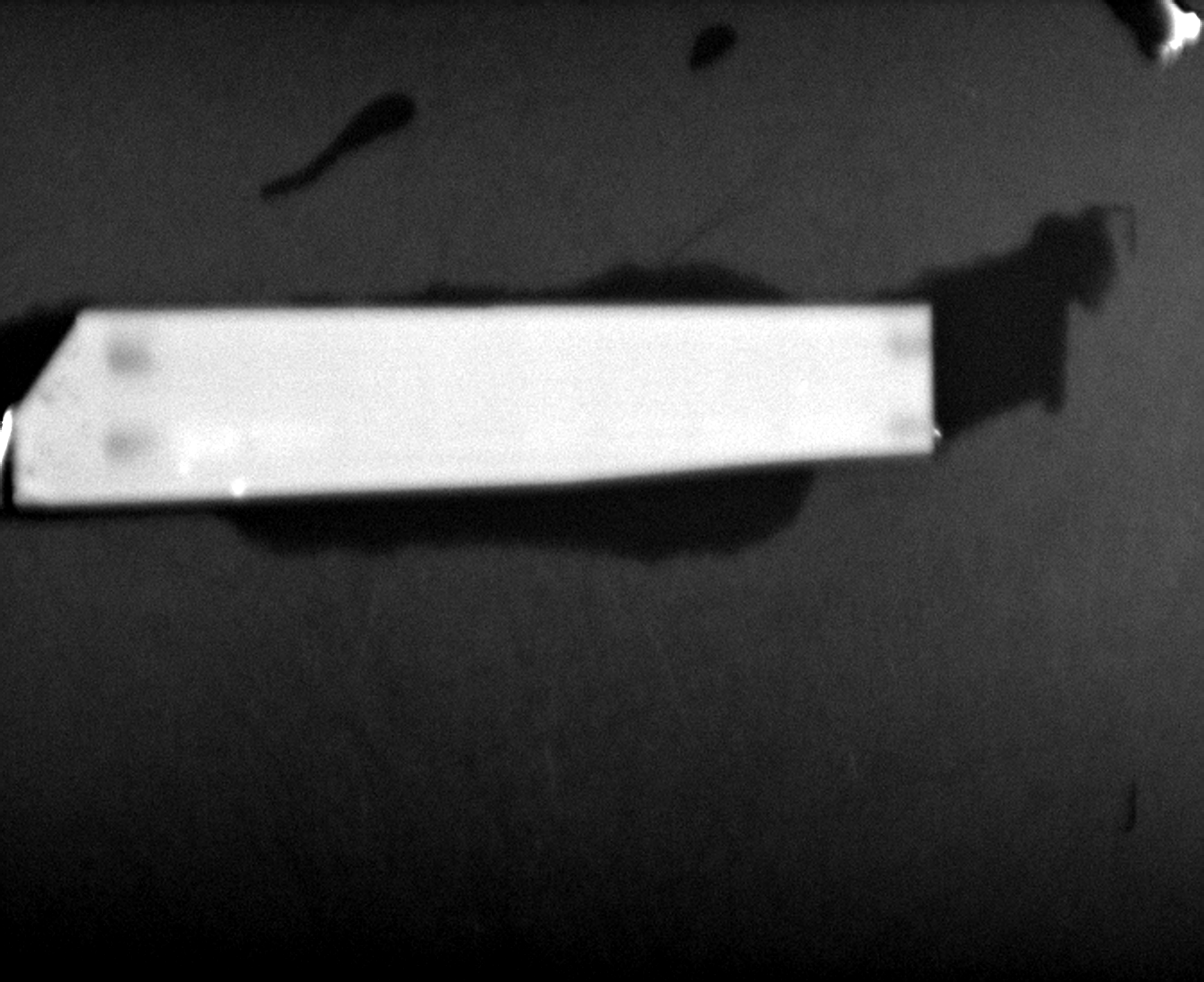


**55**

**70**

**kDa**

GAPDH


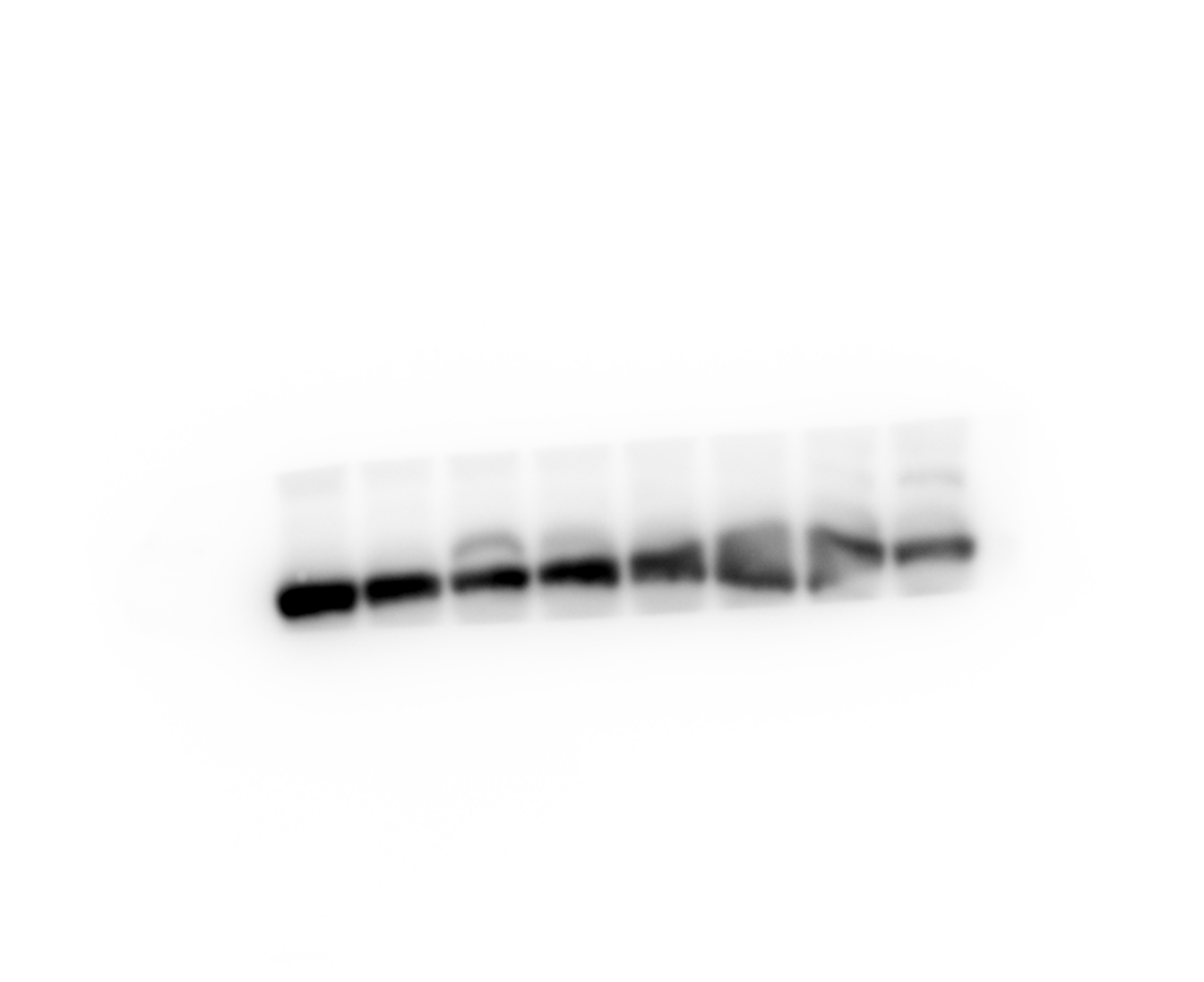


OAT1 in mouse kidney


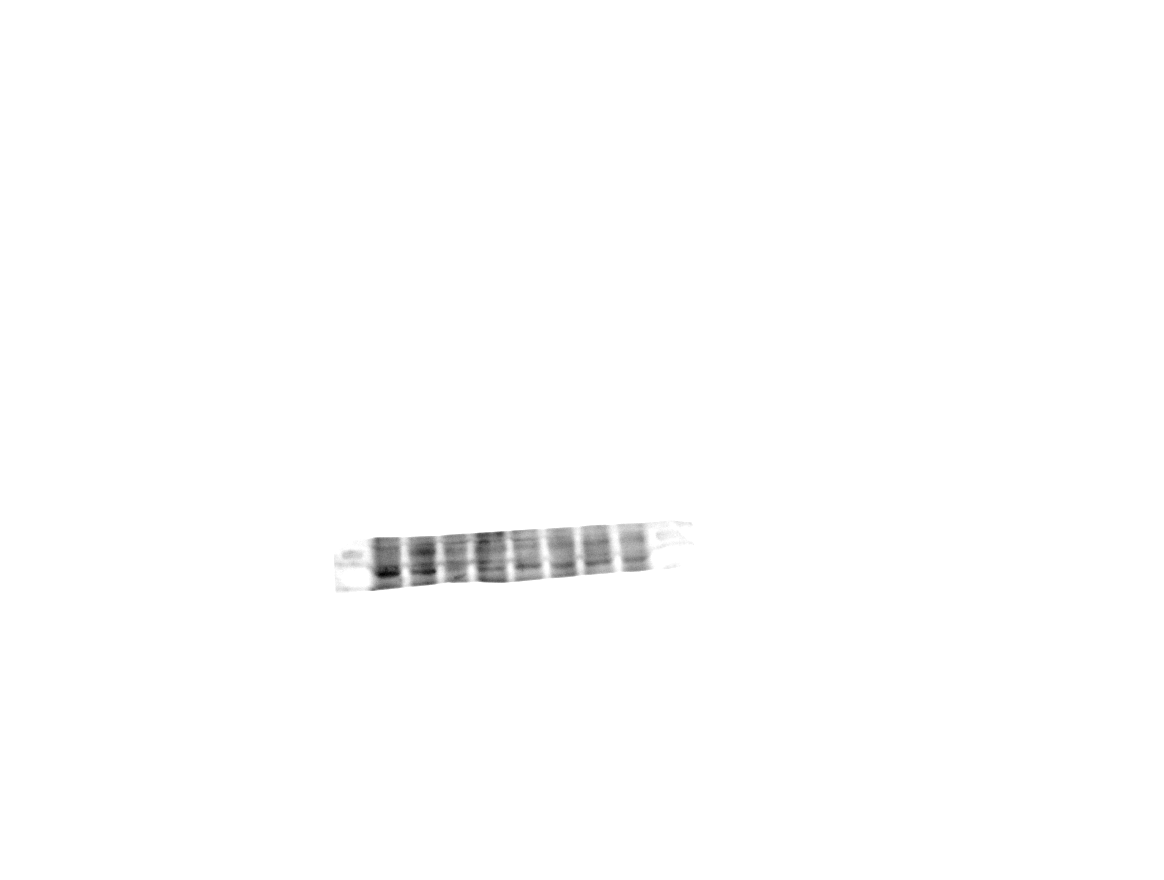


**55**

**70**

**kDa**

GAPDH


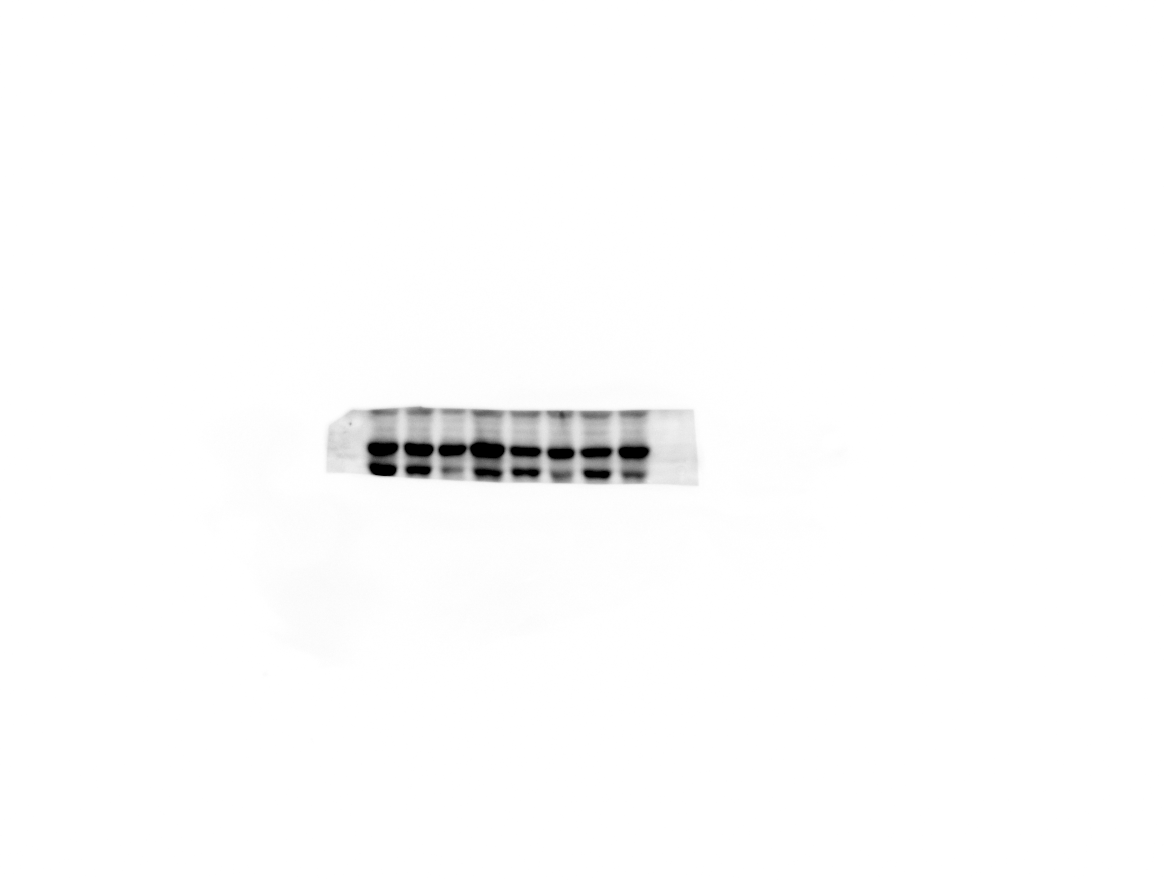


**Figure 5F**

ABCG2 in mouse colon


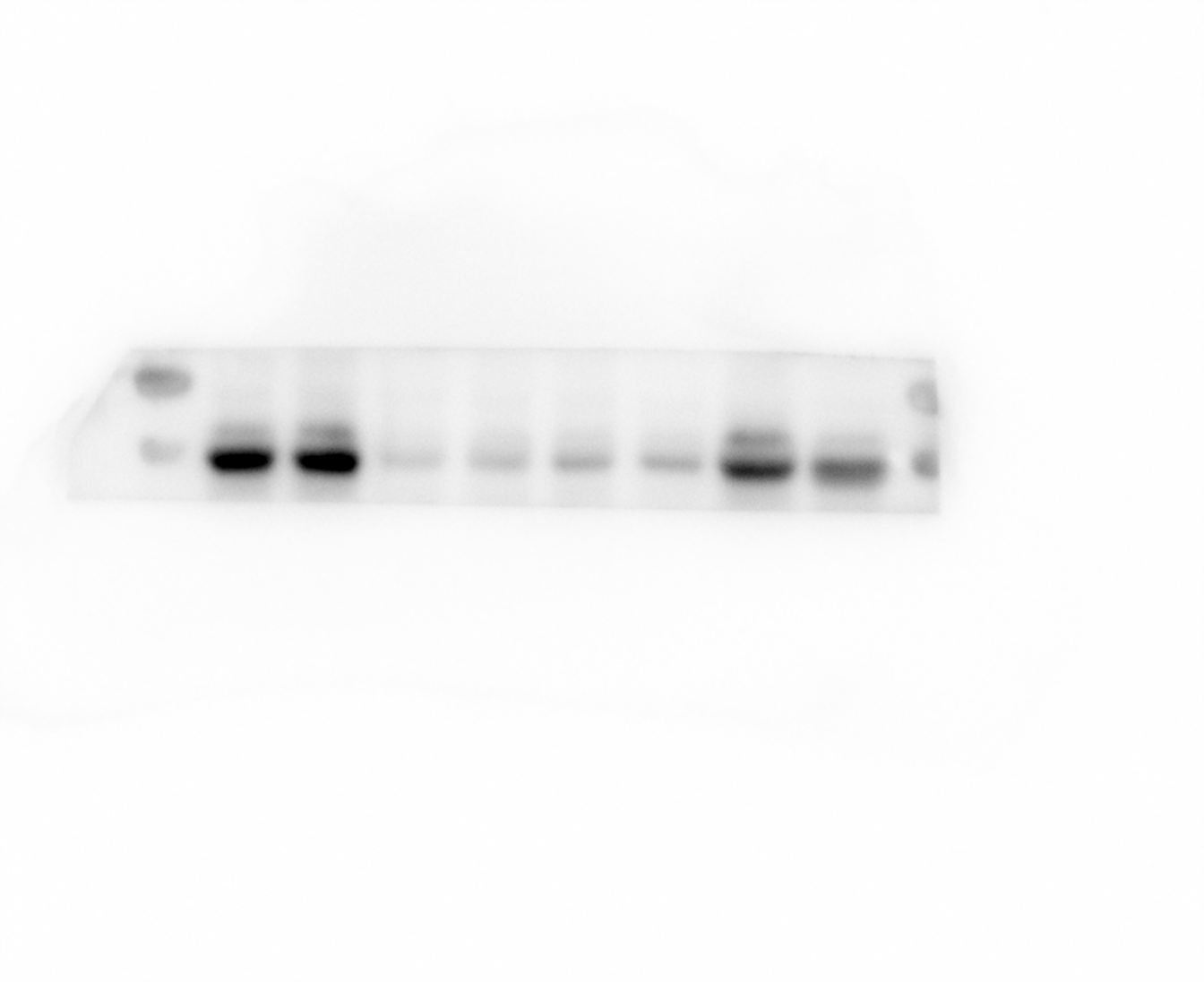


**56**

**kDa**

**70**

GAPDH


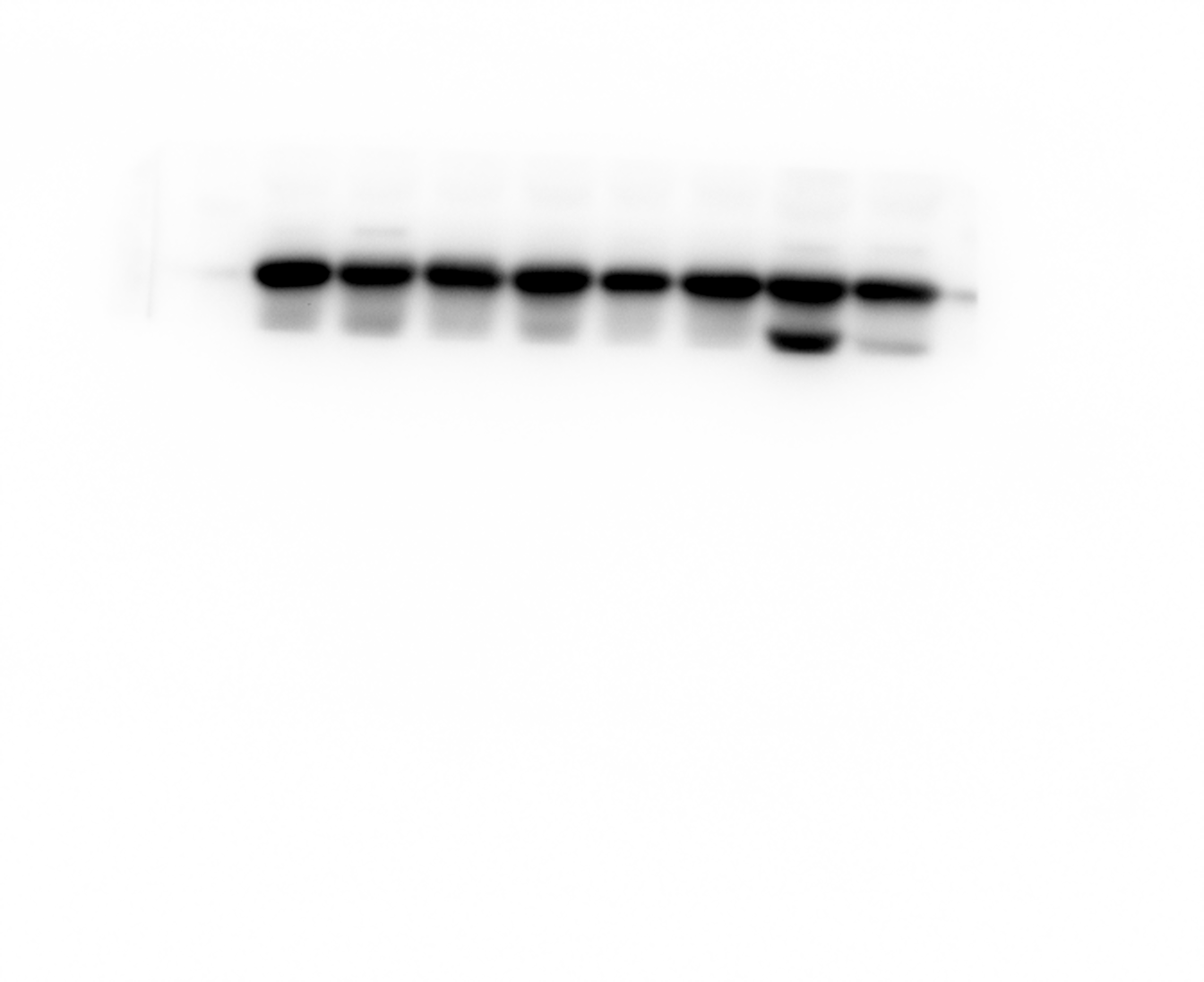


ABCG2 in mouse colon


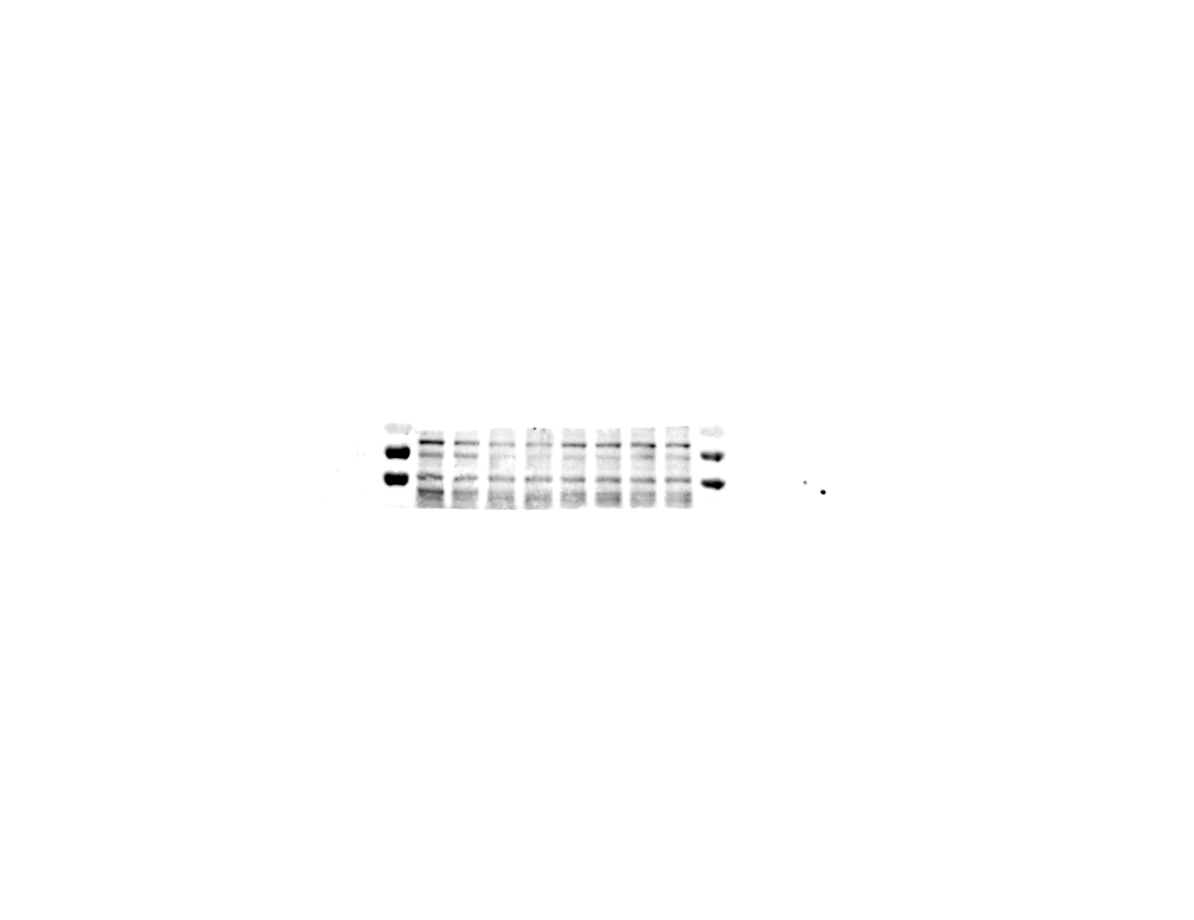


**55**

**70**

**kDa**

**56**

GAPDH


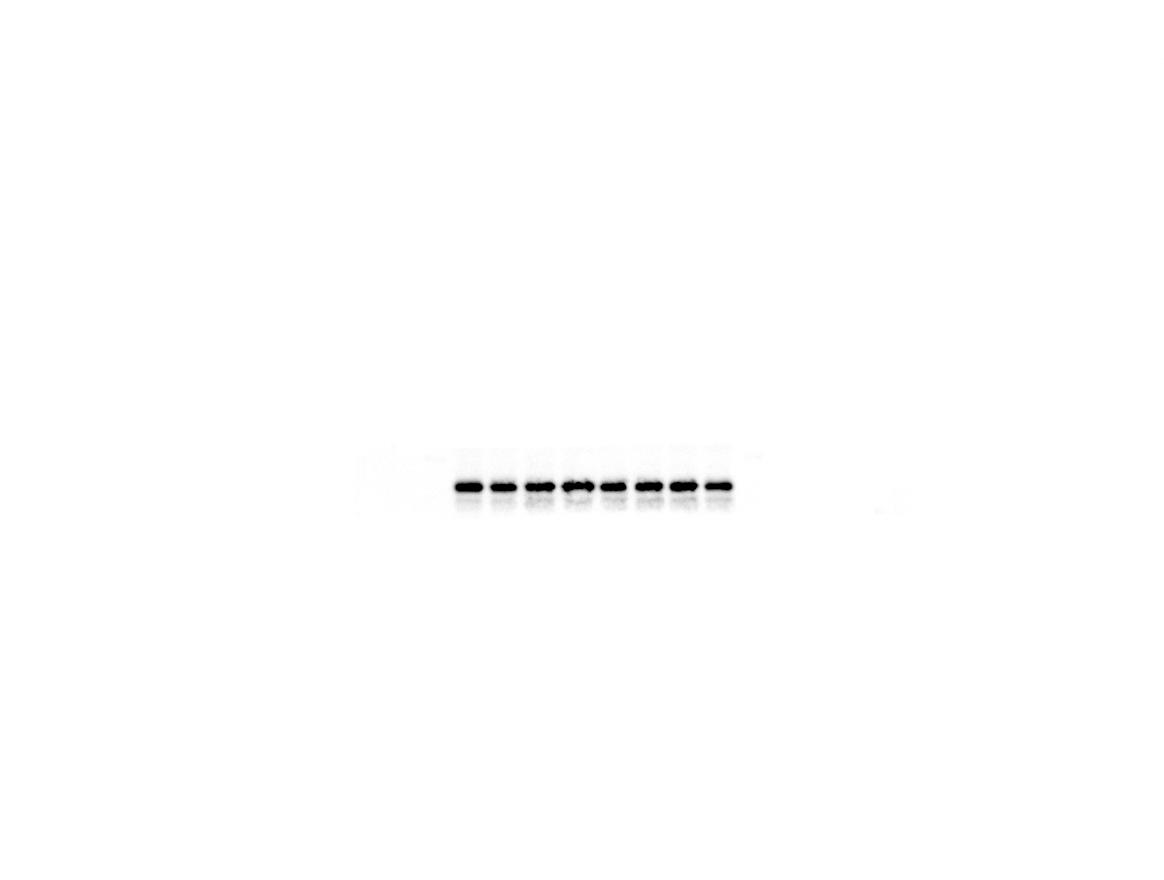


**Figure 7C**

ZO-1 in mouse colon


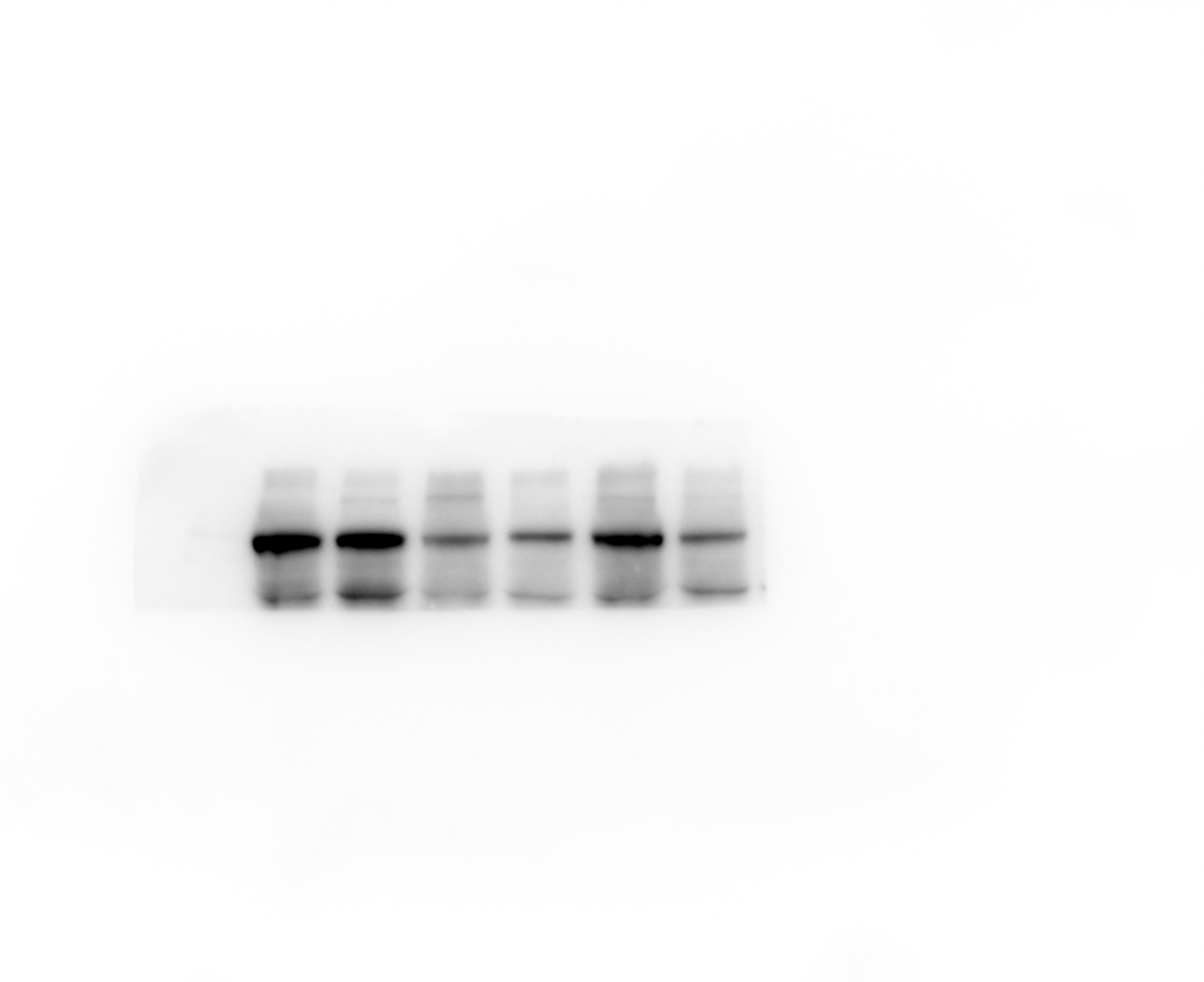


**195**

**kDa**


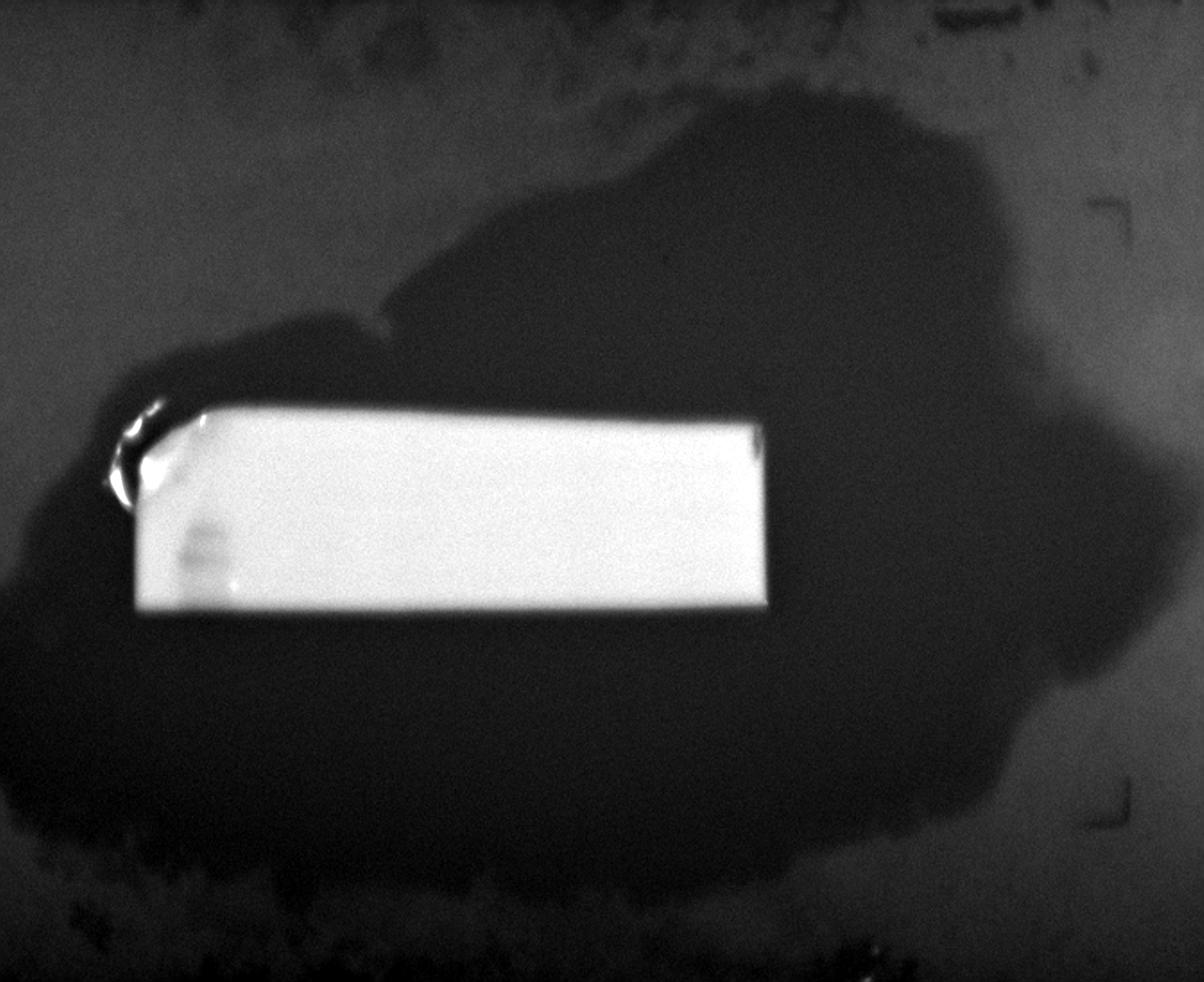


**130**

**56**

**180**

**kDa**

GAPDH


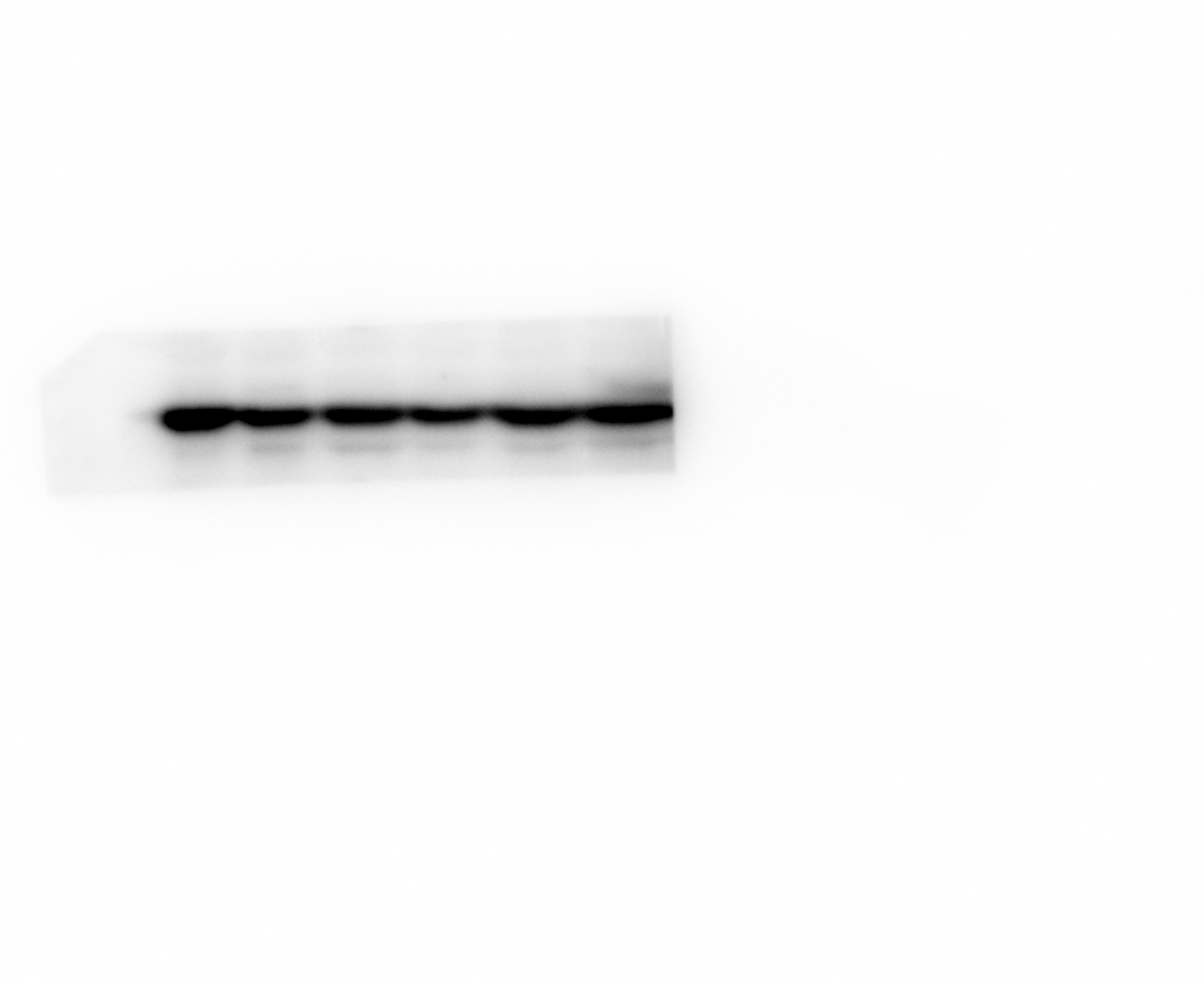


ZO-1 in mouse colon


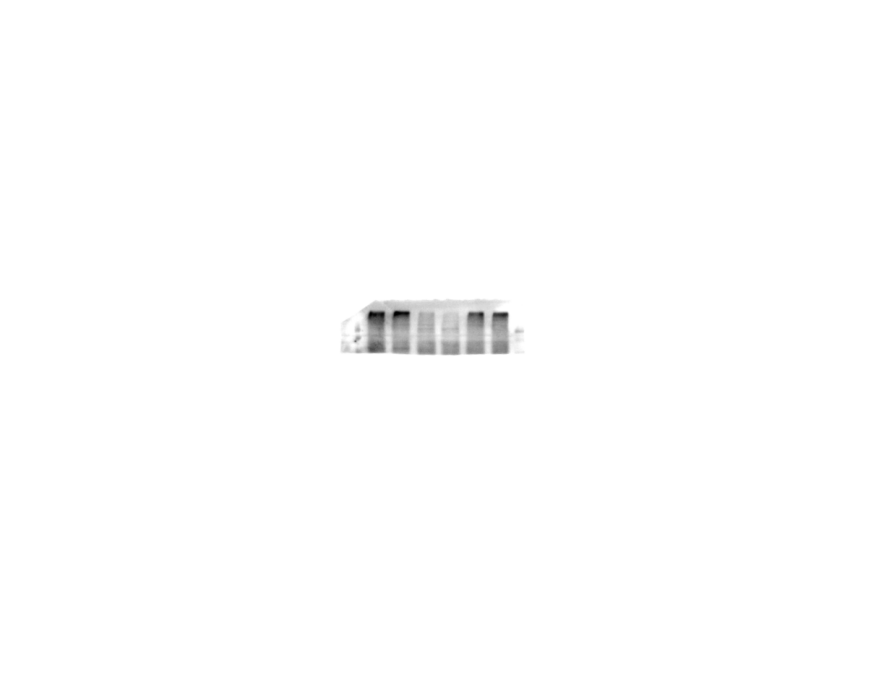


**195**

**130**

**kDa**

GAPDH


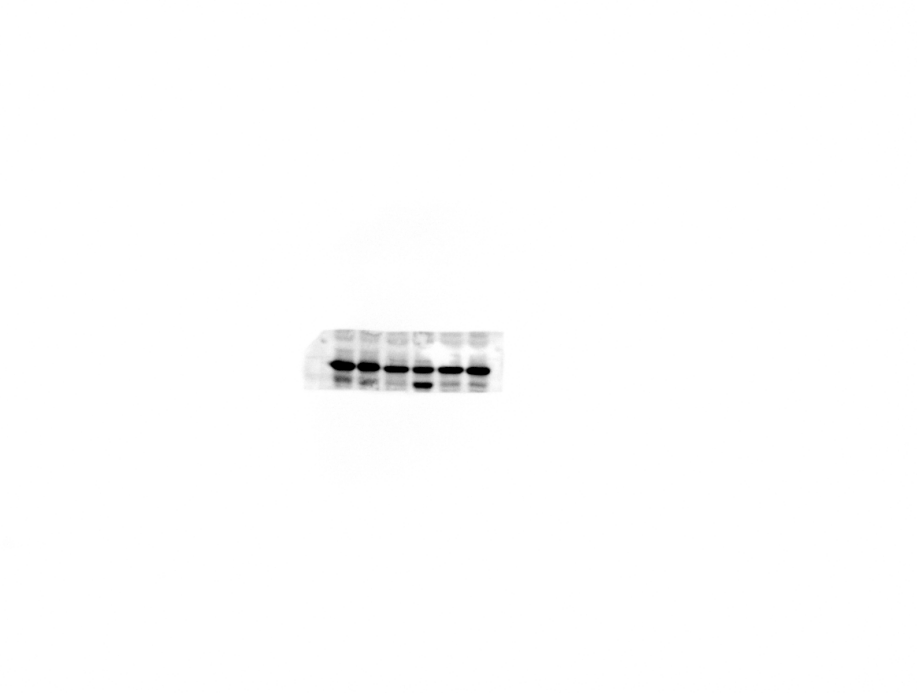


Occludin in mouse colon


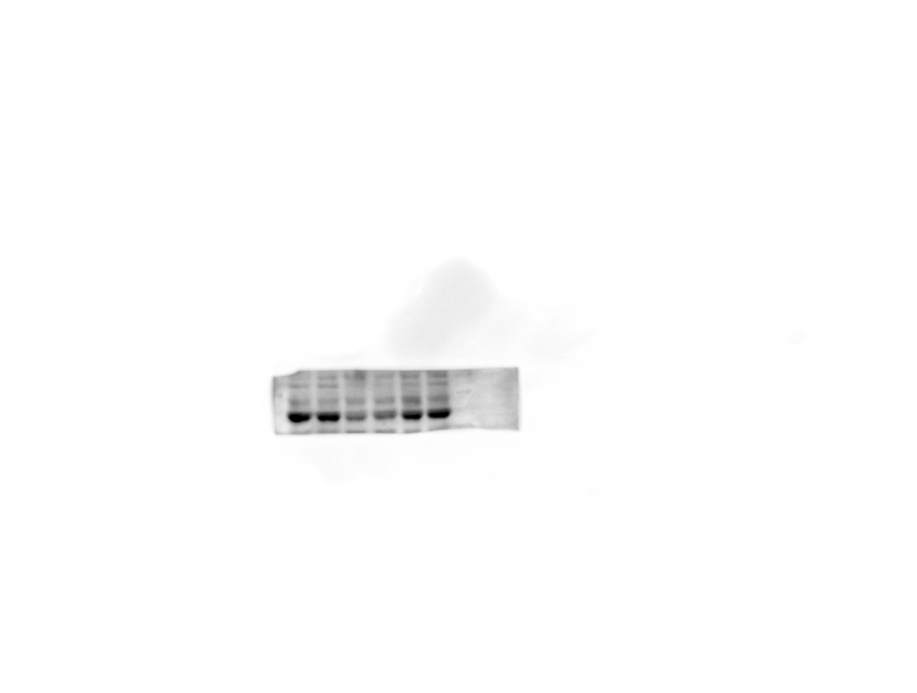


**70**

**kDa**

**59**

GAPDH


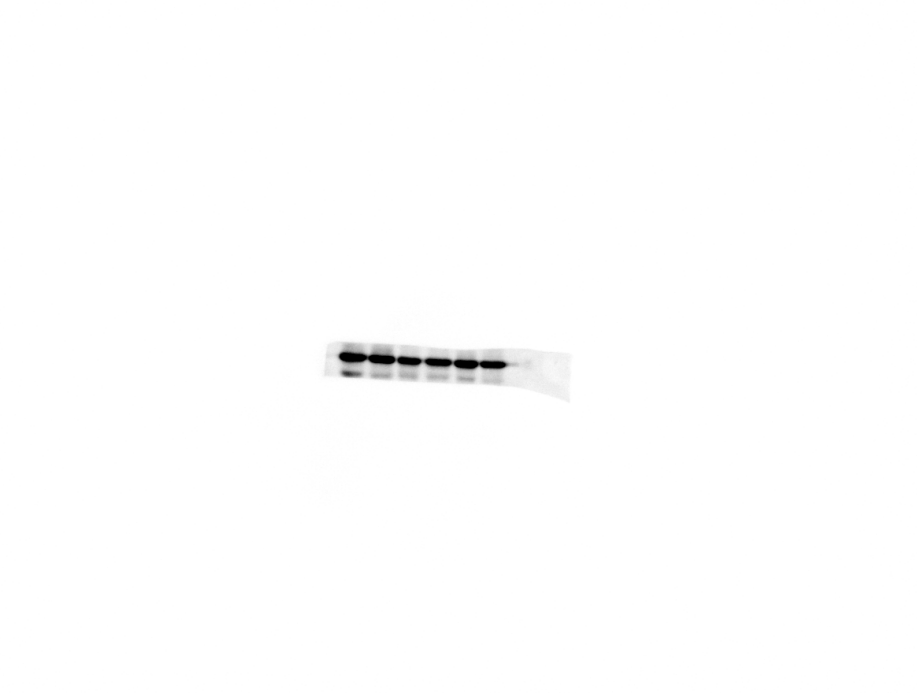


Occludin in mouse colon


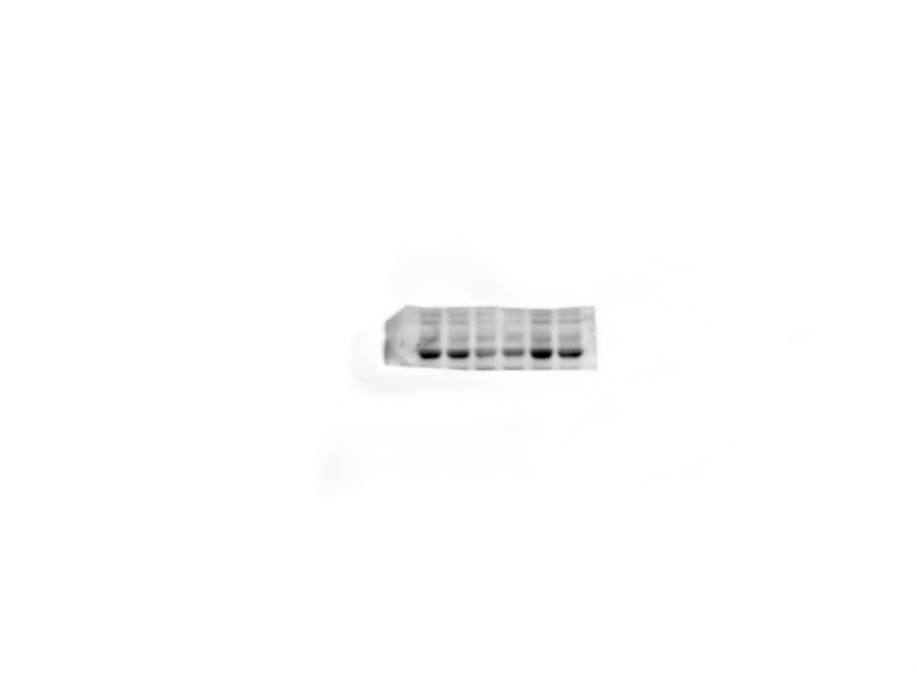


GAPDH


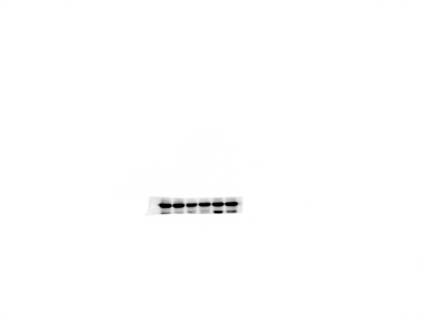

Supplement: Supplementary file 1 — Table S1: Primer sequences used in this study. Table S2: Chromatographic elution program. Table S3: Supplementary materials for the identification of major flavonoids in tfh by LC–MSn. Table S4: Bioavailability and drug‐likeness assessment of selected flavonoids based on SwissADME analysis. Figure S1: Secondary mass spectra for the identification of major flavonoids in TFH using LC–MSn. Figure S2: Three‐dimensional binding pose of MRL24 within the PPARγ binding site, with an enlarged view of the interaction environment. Figure S3: Original Western blot images. [file FSN3-14-e71925-s001.docx]
